# Dissociating reward sensitivity and negative urgency effects on impulsivity in the five-choice serial reaction time task

**DOI:** 10.1177/23982128221102256

**Published:** 2022-06-14

**Authors:** Chiara Toschi, Mona El-Sayed Hervig, Thiago Burghi, Torben Sell, Matthew Dominic Lycas, Parisa Moazen, Li Huang, Ulrik Gether, Trevor W. Robbins, Jeffrey W. Dalley

**Affiliations:** 1 Department of Psychology, Behavioural and Clinical Neuroscience Institute, University of Cambridge, Cambridge, UK; 2 Department of Neuroscience, University of Copenhagen, Copenhagen, Denmark; 3 Department of Engineering, University of Cambridge, Cambridge, UK; 4 School of Mathematics, University of Edinburgh, Edinburgh, UK; 5 Department of Physiology, Faculty of Medical Sciences, Tarbiat Modares University, Tehran, Iran; 6 Department of Physiology, Development and Neuroscience, University of Cambridge, Cambridge, UK; 7 Department of Psychiatry, Hershel Smith Building for Brain and Mind Sciences, University of Cambridge, Addenbrooke’s Hospital, Cambridge, UK

**Keywords:** Premature responding, partial reinforcement, Markov chain, frustrative non-reward, behavioural activation, dopamine

## Abstract

Negative urgency describes the tendency for rash and impulsive behaviour during negative emotional states and has been linked to a number of psychiatric disorders. However, there has been limited research on negative urgency as an explanatory mechanism for impulsivity in experimental animals. Such research has important implications for elucidating the neurobiology of negative urgency and thereby the development of future therapeutic interventions. In this study, we investigated the effects of negative urgency using a partial reinforcement schedule to increase the frequency of non-rewarded (i.e. frustrative) trials in the five-choice serial reaction time task, a widely used task to assess visual attention and impulsivity. Using a Markov chain model to analyse trial-by-trial outcomes we found that premature (i.e. impulsive) responses in the five-choice serial reaction time task were more likely to occur after a non-rewarded trial, and mostly after a previous premature trial. However, contrary to the frustration hypothesis of negative urgency, increasing the probability of reinforcement (*p*(R)) from *p*(R) = 0.5 to *p*(R) = 1 increased the number of premature responses in each session. Micro and macro levels of analyses revealed that impulsivity in the five-choice serial reaction time task is governed by at least two processes, one dependent on the overall level of reinforcement hypothesised to determine the state of behavioural activation, the second dependent on trial-by-trial outcomes consistent with negative urgency effects. These processes may depend on distinct neurobiological mechanisms and have relevance for neuropsychiatric disorders that implicate impulsive behaviours dependent on positive and negative affective states.

## Introduction

Negative urgency is regarded as an important dimension of impulsivity in humans (Cyders and Smith, 2007; Whiteside and Lynam, 2001), conceptualised as a negatively valenced level of arousal that invigorates behaviour (Eben et al., 2020). Supporting this concept, gamblers are faster to initiate future gambles after a loss than after a win (Dixon et al., 2013; Forder and Dyson, 2016; Shao et al., 2013; Verbruggen et al., 2017), while for healthy controls, scoring highly on negative urgency is associated with increased responding on trials where rewards are omitted unexpectedly (Gipson et al., 2012). In the context of psychopathology, negative urgency has been linked to unfavourable behavioural dispositions (for a review, refer to the studies by Berg et al., 2015; Smith and Cyders, 2016) including aggression (Carlson et al., 2013), problematic drug use (Latzman et al., 2013; Magid and Colder, 2007), suicidality (Nock and Prinstein, 2004; Yen et al., 2009) and eating disorders (Rosval et al., 2006; Stojek et al., 2014).

Amsel and Roussel (1952) first investigated negative urgency in experimental animals in the context of reward omission effects (ROEs). Specifically, they showed that rats ran faster to collect reinforcement in a second goal box if reinforcement in the first goal box was omitted unpredictably. ROEs have been investigated in different contexts; for example, inferred by faster response rates in both Pavlovian (Dudley and Papini, 1995) and instrumental (Gipson et al., 2012; Judice-Daher et al., 2011) tasks. However, negative urgency has been little explored as a driver for impulsivity in rats. The five-choice serial reaction time task (5CSRTT) is a widely used task to assess impulsivity and visual attention in rodents (Robbins, 2002). Trait-like impulsivity in this task, as measured by responses before the presentation of target stimuli, predicts features of addiction such as drug escalation (Dalley et al., 2007), increased propensity for relapse (Economidou et al., 2009) and compulsive drug self-administration (Belin et al., 2008).

Two previous studies found that premature responses in the 5CSRTT were more likely after non-rewarded than rewarded trials (Christakou et al., 2004; Donnelly et al., 2014). However, neither of these studies controlled for the effects of reward omission and hence negative urgency on the likelihood of a premature response. This study investigated the extent to which negative urgency, induced by omitting an expected reward during a partial reinforcement schedule, modulates the frequency of premature responses in the 5CSRTT. We specifically tested the central tenet of the frustration hypothesis, namely that frustration should ‘increase in strength as a function of non-rewarded trials’ (Amsel, 1992). Behavioural data were analysed both at the macro and micro levels. At the macro-level, ROEs were assessed for their overall impact on premature responses during each session. At the micro-level, a Markov chain approach was used to analyse behaviour on a trial-by-trial basis, specifically to investigate whether premature responses were more likely to occur after correct non-rewarded trials as opposed to correct rewarded trials. A similar model was recently used by du Hoffmann and Nicola (2016) in a study looking at approach behaviour towards a food receptacle in response to reward-predictive cues. No one, however, has used this model to look at whether trial history affects premature responses in the 5CSRTT. Compared to other methods, the (first-order) Markov chain approach has the advantage of explicitly modelling how the outcome of a trial affects behaviour in the following trial. For this reason, it is particularly suited for testing the frustration hypothesis.

We also investigated the modulation of premature responses by incentive motivational processes, specifically whether premature responses are increased by continuous rather than partial reinforcement. Such a dissociation would be consistent with an increased sensitivity to reward and heightened propensity for approach behaviour (Colder et al., 2013). Indeed the relationship between impulsivity and sensitivity to reward has long been researched in the contexts of personality (Cyders and Smith, 2007; Gray, 1987) and neuropsychiatric disorders (Luman et al., 2005; Uebel et al., 2010). For example, healthy control participants scoring highly on the ‘Non-planning Impulsiveness’ component of the Barratt Impulsivity Scale, responded more rapidly than low-impulsive individuals when the opportunity to gain rewards was highest (Cools et al., 2005). Highly impulsive rats in the 5CSRTT also generally respond faster than low impulsive rats (Toschi et al., 2021), consistent with a greater subjective utility of food reward (Niv et al., 2005). In addition, this subgroup of animals shows greater sensitivity to the reinforcing effects of cocaine (Belin et al., 2008; Dalley et al., 2007), nicotine (Diergaarde et al., 2008) and sucrose (Diergaarde et al., 2009). Evidence that motoric forms of impulsivity are more likely when reward magnitude is increased (King et al., 2016), and more pronounced in rats exhibiting an increased propensity for conditioned approach to reward-related stimuli than goal tracking animals (King et al., 2016; Lovic et al., 2011) further supports the notion that impulsivity is modulated by primary and incentive motivational processes.

Following the behavioural evidence reviewed above, the sensitivity to reward hypothesis would predict that premature responses would occur with greater frequency during a continuous reinforcement schedule. At the micro-level, the sensitivity to reward hypothesis would further predict that premature responses would be more likely after rewarded trials. However, since the receipt of reward is known to suppress behaviour through post-consummatory pausing in experimental animals (Jensen and Fallon, 1973; McHose and Gavelek, 1969; Peters et al., 2010; Seward et al., 1957) and humans (Dixon et al., 2013), it is unclear whether premature responses, which are rapid and energetically costly actions, would occur with higher likelihood after rewarded events. This study thus investigated whether premature responses in the 5CSRTT are driven predominately by positively valenced processes, consistent with the sensitivity to reward hypothesis, or negatively valenced processes predicted by negative urgency arising from frustrative non-reward.

## Methods

### Subjects

Subjects were 60 male Lister Hooded rats (Charles River, Margate, UK) weighing 280–300 g at the beginning of the experiments. Animals were acclimatised to the animal facility under a 12 h:12 h light cycle (lights off at 7 a.m.) for a minimum of 7 days before any procedure began. When rats reached a body weight of approximately 300 g, they were food-restricted to maintain approximately 90% of their free-feeding weight (19 g of Purina rodent chow per animal and day; adjusted for reward pellet consumption during testing). Water was available ad libitum and food was given at the end of each day’s testing. All procedures conformed to the UK (1986) Animal (Scientific Procedures) Act (Project licence 70/7548 and PA9FBFA9 F: Neurobehavioural mechanisms of mental health, held by Dr A. L. Milton) and were approved by the local Ethics Committee at Cambridge University. Rats were housed in groups of four. Two cohorts of rats were used for this study for replication purposes: the first consisted of 24 animals, the second consisted of 36 animals. The sample size was chosen based on previous studies in the lab using the 5CSRTT.

### 5CSRTT task

#### Apparatus

Twelve five-hole operant chambers (Med Associates, Georgia, VT) controlled by two computers and Whisker Control software (Cardinal and Aitken, 2010) were used. Each chamber was enclosed in a ventilated sound-attenuating box, fitted with five apertures in a curved wall and a food magazine on the opposite wall of the box that delivered rodent sugar pellets (TestDiet^®^, Purina, UK). A yellow light-emitting diode (LED) stimulus was placed at the rear of each aperture. The food magazine and entire chamber was illuminated by LEDs. Infrared beams detected responses in the magazine and apertures.

#### Behavioural training

All rats were trained in the 5CSRTT as described previously (Bari et al., 2008). Animals were trained to detect a brief visual cue appearing in one of five apertures of the operant chambers. Trials were initiated when the rat made a response into the food magazine. After 5 s, a visual cue was in one of the five open apertures. A response was deemed ‘correct’ if the animal poked into the hole where the light was presented within 5 s of target presentation. A nose-poke response occurring before the appearance of the visual cue was considered ‘premature’, while a response occurring in any of the apertures where the light was not presented was considered ‘incorrect’. A failure to respond within 5 s of target presentation was recorded as an ‘omission’. Only correct responses were rewarded with a food pellet (Noyes dustless pellets, Research Diets, UK), while incorrect, premature and omission responses were punished with a time-out period of 5 s. During a time-out period, the animal was required to wait for the beginning of the next trial to engage again with the task. Nose-pokes in any of the holes made after a correct or incorrect response, but prior to reward collection, were deemed ‘perseverative’ but were not signalled by punishment. Each session lasted a maximum of 100 trials or 30 min, whichever occurred first. The stimulus duration was initially 30 s but was gradually reduced until animals reached stable baseline performance (accuracy, > 80% correct choice and < 20% errors of omission). Rats in Cohort 1 (*N* = 24) were trained to reach a stable baseline performance on the 5CSRTT with a final stimulus duration of 0.6 s and an inter-trial interval (ITI) of 5 s. Rats in Cohort 2 (*N* = 36) were trained to reach a stable baseline performance on the 5CSRTT with a final stimulus duration of 0.7 s and an ITI of 5 s. Each session lasted a maximum of 100 trials or 30 min, whichever limit was reached first.

Following task acquisition, a variable inter-trial interval (vITI) session was introduced, which consisted of a pseudo-random presentation of trials with 3 s, 5 s, 7 s and 9 s ITI. Each ITI was presented at least 50 times and the session ended when animals had completed 200 trials or after 2 h (whichever occurred first). Animals were then screened for impulsivity during three vITI challenge sessions, with 1 day between each that consisted of the standard fixed ITI of 5 s. Premature responses across the vITI challenge sessions were averaged and the upper (i.e. the highest-impulsive rats, HI) and lower quartiles (i.e. the lowest-impulsive rats, LI) were selected. Animals falling between these two extremes were classified as mid-impulsive (MID) rats.

#### Experiment 1

Rats were tested on the 5CSRTT using a partial reinforcement schedule with probabilities of reinforcement *p*(R) of 0.2, 0.5, 0.8 and 1 (stimulus duration: 0.6 s; ITI 5s; time-out 5 s). In this schedule, ‘correct’ responses were rewarded with probability *p*(R). Consequently, five types of trial outcomes were possible: ‘correct rewarded’ (R), ‘correct non-rewarded’ (NR), ‘incorrect’, ‘omission’ and ‘premature’. Individual sessions consisted of a fixed reward probability and were presented using a Latin square design to prevent order effects. Between each session, rats experienced a single session with continuous reinforcement (i.e. *p*(R) = 1). Reinforcement of correct trials was pseudo-randomised such that every 20 trials rats were exposed to all the planned rewarded and non-rewarded contingencies according to the probability of each specific session (*p*(R) = 0.2, 0.5, 0.8 or 1), as determined by the Latin square design. The probability of reinforcement only changed between sessions and not within a session.

#### Experiment 2

To test how premature responses are affected by partial reinforcement in conditions of increased waiting time (i.e. ITI) and of reduced time-out punishment (TO), rats were then tested on both continuous (*p*(R) = 1) and partial reinforcement (*p*(R) = 0.5) schedules with systematic variations in the ITI (7 s versus 5 s) and TO (5 s versus 1 s). Sessions were presented using a Latin square design with 5 days of baseline testing between each variation to avoid habituation.

#### Markov chain model

To investigate whether premature responses occurred more frequently after NR trials, we estimated a first-order discrete-time Markov chain model. A discrete-time Markov chain is a stochastic model describing the evolution of a random sequence of states $X_{t}$ , where t is an integer (Davison, 2003). In this case, for each experiment, the state *$X_{t}$* represents the type of trial outcome (R, NR, premature, incorrect or omission) observed at trial t within a session. For instance, if the 5th trial is a premature response, then $X_{5}=\mathrm{premature}$ . In a first-order Markov chain, the value of the state $X_{t}$ given the previous state $X_{t-1}$ is conditionally independent of the states preceding $X_{t-1}$ , that is

$$P\left(X_{t}|X_{t-1},X_{t-2},\ldots ,X_{0}\right)=P\left(X_{t}|X_{t-1}\right)$$

The conditional probability $P(X_{t}|X_{t-1})$ describes the probability that the state $X_{t-1}$ is followed by $X_{t}$ and is called a transition probability. If all transition probabilities are constant across time, the Markov chain is called homogeneous. For a homogeneous Markov chain, the set of all possible transition probabilities between states can be summarised in a constant probability matrix, the entries of which can be estimated from the transition frequencies observed during a session of an experiment. For instance, for any trial t , we can estimate the probability that an NR response is followed by a premature response using

$$P\left(\begin{array}{l} X_{t}=\mathrm{premature}|X_{t-1}\\ \;\;\;\;\;\;\;\;=\mathrm{NR} \end{array}\right)=\frac{\#NR\;\mathrm{followed}\;\mathrm{by}\;\mathrm{premature}}{\#\;\mathrm{total}\;\mathrm{NR}\;\mathrm{responses}}$$

**Figure 1.**
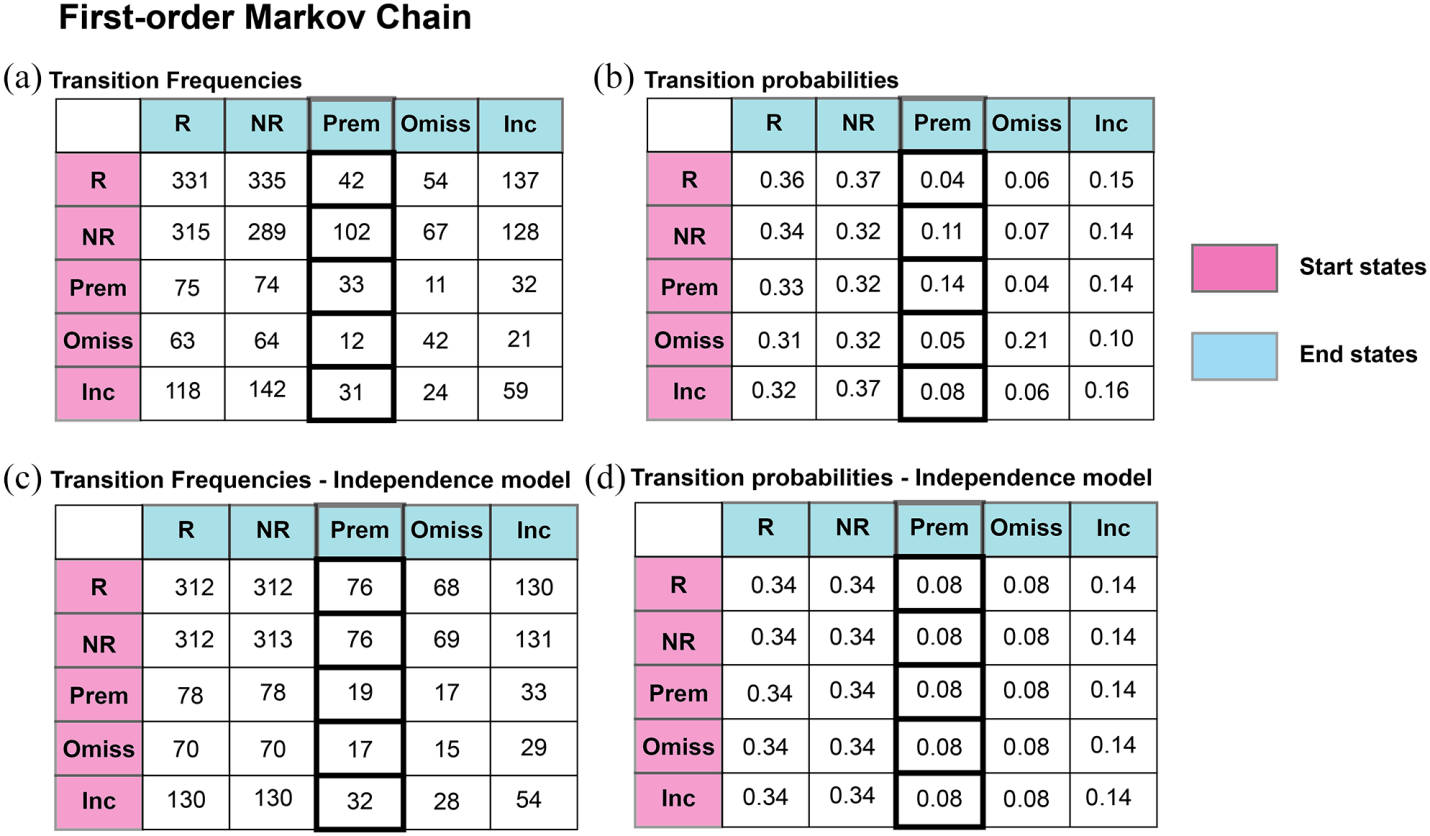
Representation of the (a) transition frequency matrix and transition probabilities matrix estimated from a first order Markov-chain on the performance of the 5CSRTT. Representation of the independence model with (c) transition frequencies and (d) transition probabilities fitted based on the raw (observed) data in (a). The total number of trials considered in (a) is 2601. Fitted data are the transition frequencies and probabilities that would be observed if there were no dependencies between trials. The pink column represents starting states, whereas the blue row represents end states. The column with margins in bold in (a), (b) and (c) captures the frequencies (a and c) and probabilities (b) of one-step transitions leading to a premature response. Data from a session with 5 s ITI, 5 s TO and *p*(R) = 0.5 are considered in this graph.

To illustrate these calculations, the observed transition frequencies from a sample session of Experiment 1 are summarised in Figure 1(a), and the first-order transition probabilities estimated with those frequencies are summarised in Figure 1(b). To test the hypothesis that the observed transition frequencies are explained by a first-order Markov chain, we also consider a zeroth-order Markov chain model, also referred to as an independence model, where the state at trial t is independent of all the preceding trials, that is

$$P\left(X_{t}|X_{t-1},X_{t-2},\ldots ,X_{0}\right)=P\left(X_{t}\right)$$

In particular, transition probabilities under the zeroth-order model are independent of preceding states. For instance, we have

$$P\left(X_{t}=\mathrm{premature}|X_{t-1}\right)=P\left(X_{t}=\mathrm{premature}\right)$$

regardless of the value of $X_{t-1}$ . For a homogeneous independence model, these probabilities are constant with respect to t , and can also be estimated from the transition frequencies observed during a session. For instance, for any trial t , we can estimate the probability of a premature response using

$$P\left(X_{t}=\mathrm{premature}\right)=\frac{\#\mathrm{total}\;\mathrm{premature}\;\mathrm{responses}}{\#\;\mathrm{total}\;\mathrm{responses}}$$

Figure 1(d) summarises the zero-order transition probabilities estimated using the transition frequencies of Figure 1(a). Using these probability estimates, we can compute the expected transition frequencies between states under the zeroth-order Markov chain. For instance, the expected transition frequency from NR to premature responses is given by

$$\begin{array}{l} \begin{array}{c} E\left[\#\mathrm{NR}\;\mathrm{followed}\;\mathrm{by}\;\mathrm{premature}\right]=P\left(X_{t}=\mathrm{premature}\right)\times P\left(X_{t-1}=\mathrm{NR}\right)\times \left(\#\mathrm{total}\;\mathrm{responses}\right) \end{array}\\ \;\;\;\;=\frac{\left(\#\mathrm{total}\;\mathrm{premature}\;\mathrm{responses}\right)\times \left(\#\;\mathrm{total}\;\mathrm{NR}\;\mathrm{responses}\right)}{\left(\#\mathrm{total}\;\mathrm{responses}\right)} \end{array}$$

This is illustrated in Figure 1(c). The above equation shows that the expected transition frequencies under the independence model can also be computed based on observed transition frequencies (Figure 1(a)).

#### Statistical analysis

The dependent variables of interest were: latency to make a correct response; number of correct responses; number of omissions; latency to start a new trial after a period of time-out (defined as the time it takes an animal to poke into the food magazine to initiate a new trial after a time-out punishment), and the number of premature responses. Statistical tests were performed using RStudio, version 1.2.1335 (RStudio, Inc). Data were subjected to Linear Mixed-Effects Model (LMEM) analysis with the lmer package in R. All models with a within-subject factor had the factor ‘subject’ modelled as a random slope to account for individual differences between rats across testing sessions. When significant three-way interactions were found, further analyses were performed by conducting separate multilevel models on a specific variable of interest. For all analyses, significance was considered at α = 0.05. When significant interactions were found, post hoc Tukey’s tests were used with corrected pairwise comparisons. In all experiments, the number of correct responses and of premature responses were square root transformed and latencies were log-transformed to satisfy the assumptions of normally distributed datasets. Additional information on model parameters, such as: coefficient estimates, standard error (SE) of the coefficients and *t* values, are reported in Tables S4–S6 in the supplementary material. To visualise the temporal development of the probability of making any response type within a session, a multinomial logistic regression was fit to the performance, for each experiment, of the session with *p*(R) = 0.5 (see Figure S1 in the supplementary material).

The appropriateness of the first-order Markov chain model was tested against an independence model, the zeroth-order Markov chain, which one would observe if there was no history dependence in the state transitions. To test this, the likelihood ratio statistic W was calculated using the observed and expected transition frequencies under the independence model (Davison, 2003). This statistic approximates Pearson’s χ^2^ statistic and follows asymptotically a χ^2^ distribution with (*S* − 1)^2^ degrees of freedom (DOFs), where *S* denotes the number of states in the matrix. In this case, the χ^2^ distribution had 16 DOFs. To reject the zeroth order model, the *W* statistic should be greater than the α significant point of the χ^2^ distribution with the DOFs considered in each specific test. Alpha was set at 0.05. Therefore, the zeroth order model was rejected when *W* was greater than the critical value 26.30 (based on the χ^2^ distribution with 16 DOFs).

To represent visually the extent to which each transition probability leading to a premature response deviated from the independence model, the variable *Y* = (O − E)/E^1/2^ was calculated (Davison, 2003, Chapter 6) for each start state ending in a premature response and plotted in bar graphs where the origin of the *x*-axis represents no deviation from the independence model (see Figures 3(b); 7; and S4b). Here, O stands for ‘observed’ and indicates the number of trial types (or transition frequencies) that the animals made in each specific condition (for example, each cell of Figure 1(a)). E stands for ‘expected’ and represents the expected number of trial types (or expected transition frequencies) that the animals would make in each specific condition under the independence model, (for example, each cell of Figure 1(c)). We focused mainly on transition probabilities between trials in the partial reinforcement (*p*(R) = 0.5) condition, as only this condition has an equal number of R and NR trials and thus ensures a similar number of frustrative and non-frustrative events. We did, however, also look at transition probabilities leading to a premature response in the other schedules of reinforcement (*p*(R) = 0.2; *p*(R) = 0.8; *p*(R) = 1) and these are reported in the supplementary materials (Figures S5 and S6 in the supplementary material). To test whether the first-order Markov Chain was homogeneous, we split each session into two halves and estimated the transition probabilities assuming homogeneity within each half. Figures S2 and S3 illustrate the estimates for the partial reinforcement condition *p*(R) = 0.5. Table S1 describes the diagnostic tests applied to the first-order Markov chains estimated on the first and second halves of each 5CSRTT session in each manipulation. Given that differences in the transition probabilities leading to a premature response between the two halves of each session were small, we performed all other analyses assuming homogeneity of the Markov chain for the whole session. This was done to increase the statistical robustness of the diagnostic tests.

Further tests were applied to test whether transitions that led to a premature response deviated from the independence model. To achieve this, we used standard asymptotic theory for multinomial or normal distribution χ^2^ (as explained by Anderson and Goodman, 1957) with 5 DOFs. Here, the independence model was rejected when χ^2^ was greater than the critical value of 11.07 (based on the χ^2^ distribution with 5 DOFs).

## Results

### Experiment 1

To investigate whether premature responses were modulated by the level of motivation (sensitivity to reward hypothesis), it was first necessary to evaluate the extent to which partial reinforcement affected the motivation of animals to engage with the task. This was achieved by analysing the following motivational variables: latency to make a correct response, number of correct responses, number of omissions and the latency to start a new trial after a period of time-out. The latency to make a correct response was further analysed by comparing R versus NR trials to test whether animals could predict whether an upcoming trial was rewarded or not. Experiment 1 was conducted on two separate cohorts of animals to test for replicability of findings. Findings for cohort 1 are reported below, whereas findings for cohort 2 are reported in the supplementary section (Figure S4).

### Effects of partial reinforcement on 5CSRTT performance

**Figure 2.**
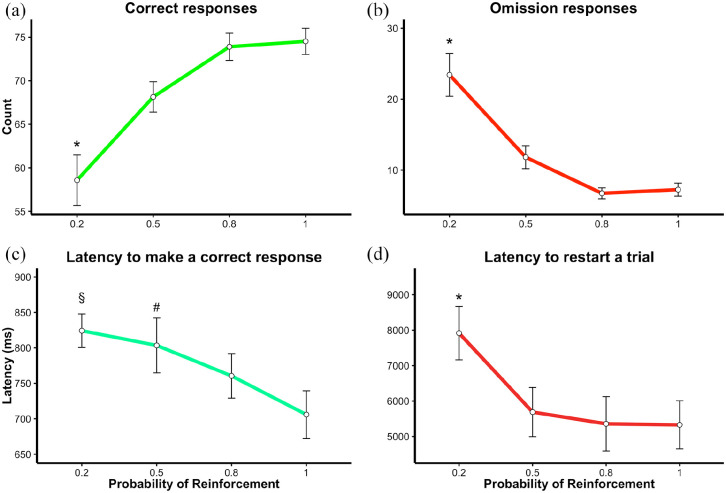
Experiment 1. Effect of reinforcement rate on different indices of motivation. (a) Correct responses, (b) omission responses, (c) latency to make a correct response (ms), and (d) latency to start a trial (ms). Means and standard error (SE) are reported. *Statistical difference between *p*(R) = 0.2 and all other schedules of reinforcement, *p* < 0.05. ^§^Statistical difference between *p*(R) = 0.2 versus *p*(R) = 1, *p* < 0.05. ^#^Statistical difference between *p*(R) = 0.5 versus *p*(R) = 1, *p* < 0.05. LMEM was used for this analysis.

Figure 2 shows the effects of partial reinforcement on the number of correct and omission responses, latency to make a correct response and latency to re-start a trial, across sessions with different reinforcement rates. For correct responses, there was a main effect of reinforcement rate [F(3,54) = 15.33, *p* < 0.001], with rats making significantly fewer correct responses with *p*(R) = 0.2 compared to all the other reinforcement rates (*p* < 0.01 for all comparisons). For omissions, there was a main effect of reinforcement rate [F(3,54) = 25.62, *p* < 0.001], with rats making more omissions with *p*(R) = 0.2 compared to all the other reinforcement rates (*p* < 0.001 for all comparisons). Reinforcement rate also influenced the latency to make a correct response [F(3,54) = 6.95, *p* < 0.001]. Specifically, animals were slower with *p*(R) = 0.2 and *p*(R) = 0.5 compared with *p*(R) = 1 (*p* < 0.01). We also found a main effect of partial reinforcement on the latency to start a trial after a time-out, [F(3,53.34) = 5.46, *p* = 0.002]. Specifically, animals were slower when *p*(R) was = 0.2 compared to all other reinforcement rates (*p* < 0.05 for all comparisons). Latency to make an R response was analysed separately from latency to make an NR response, to test whether rats could predict which correct response was going to be rewarded. Analyses revealed that trial outcome did not affect latency to make a correct response [F(1,90) = 0.13, *p* = 0.715].

As expected, and in line with previous research (Mohebi et al., 2019), partial reinforcement had an effect of motivation to engage with the task, with rats being slower to make a correct response with decreasing reinforcement rates. Other measures that changed with decreasing reinforcement rates were (1) lower number of correct responses, (2) higher number of omissions and (3) slower latencies to initiate a new trial after a time-out. Crucially, when making a correct response, rats could not predict whether the trial would be rewarded or not, as shown by identical latencies for R and NR trials.

### Effects of partial reinforcement on premature responses

**Figure 3.**
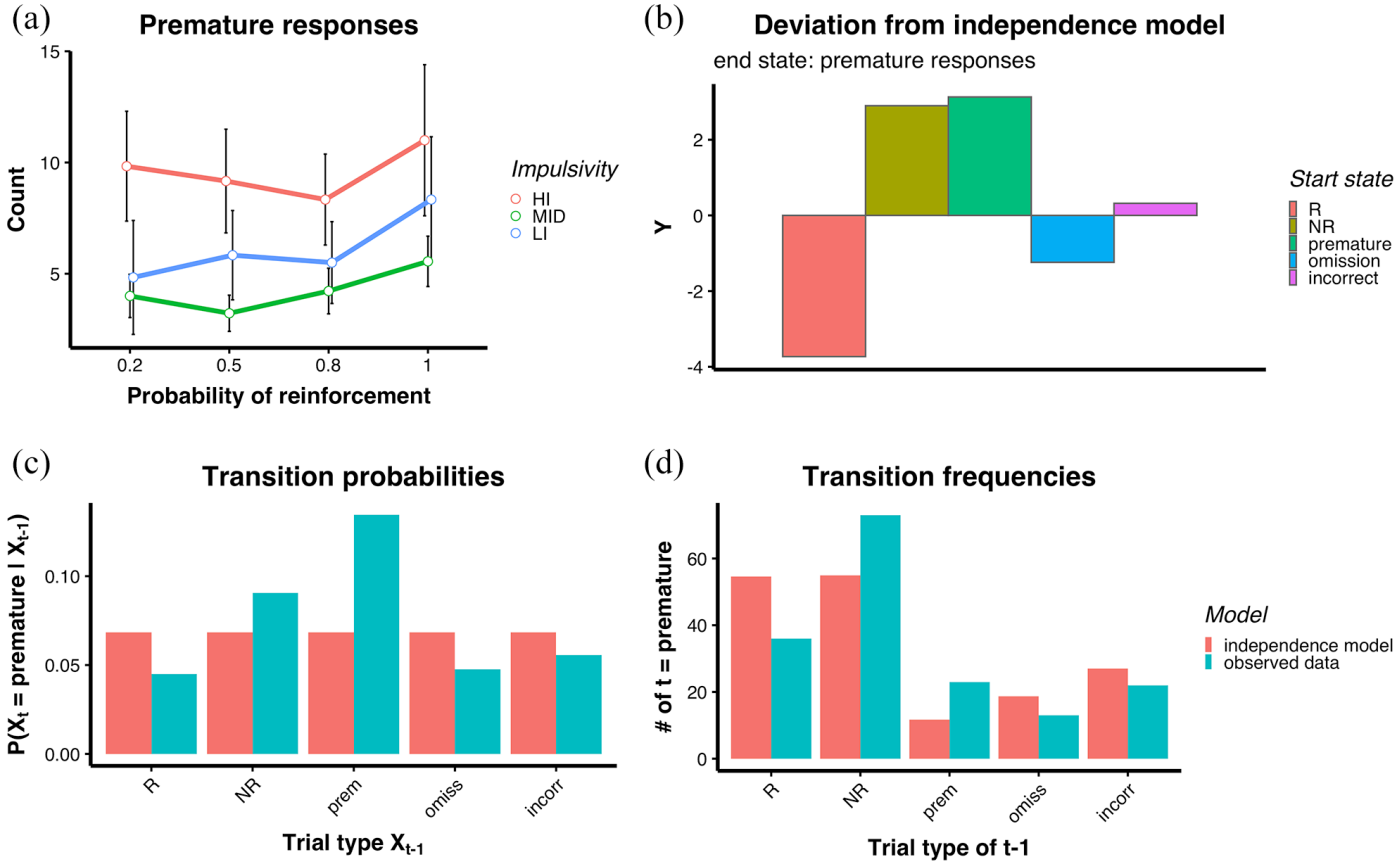
Experiment 1. (a) Effects of reinforcement rate on premature responses (Mean and SE); LMEM was used for this analysis. (b) Deviation from the independence model of transition probabilities leading to premature responses for Experiment 1 on Cohort 1 during *p*(R) = 0.5. *Y* = (O − E)/E^1/2^ was calculated for each start state ending in a premature response, O = observed data and E = expected data under the assumption of the independence model. The value 0 on the *x*-axis represents no deviation from the independence model. (c) Transition probabilities leading to a premature response (end state). *Y*-axis shows the probability to transition to a premature response as an end state (trial t). *X*-axis shows starting states (trial *t*−1). (d) Frequencies of one-step transitions leading to a premature response (end state). *Y*-axis shows how often a premature response was an end state (trial *t*). *X*-axis shows starting states (trial *t*−1). Red = independence model; Blue = observed data.

Having established that partial reinforcement modulates motivation to engage with the task, it was important to test whether changes in motivation affected the frequency of premature responses, in line with the sensitivity to reward hypothesis. Figure 3(a) shows the effects of partial reinforcement on premature responses. Animals tended to make more premature responses during sessions with *p*(R) = 1 compared with other reinforcement rates; however, this difference was not significant [main effect of *p*(R): F(2,18) = 3.25, *p* = 0.062]. However, significant negative correlations were found between premature responses and correct response latencies (*p*(R) = 0.2: *r* = −0.50, *p* = 0.022; *p*(R) = 0.5: *r* *=* −0.49, *p* = 0.024; *p*(R) = 0.8: *r* = −0.46, *p* = 0.036; *p*(R) = 1: *r* = −0.45, *p* = 0.041).

### Consequences of a rewarded or non-rewarded trial on premature responses

Transition probabilities between trial types were analysed to evaluate whether animals were more likely to make a premature response after a frustrative event, such as an NR trial. A *W* statistic of 73.61 indicated that the Markov chain model violated the independence model with a significance level of *p* < 0.001, indicating that the probability to transition to a state *t* did depend on the previous state *t*−1. A χ^2^ test run on the frequencies of one-step transitions leading to a premature response showed that these were significantly different from the distribution that would be expected if there were not dependencies between trials, χ^2^ = 33.71, *p* < 0.001 (under the χ^2^ distribution with 5 DOFs). Figure 3(c)–(**d)** show how the transition probabilities (Figure 3(c)) and frequency (Figure 3(d)) leading to a premature response deviated from the independence model. The largest deviations from the independence model were a lower-than-expected probability to transition to a premature response from an R trial (*Y* = −3.73), and a higher-than-expected probability to transition to a premature response from a premature response (*Y* = 3.13, as shown in Figure 3(b)). Rats were also more likely to make a premature response after an NR trial (*Y* = 2.90, see Figure 3(b)), compared to what would be expected under the independence model. Results for the consequences of a rewarded or non-rewarded trial on premature responses for all the other schedules of reinforcement are shown in the supplementary Figures S5 and S6.

### Interim summary

A Markov chain model revealed that there are dependencies between trial types, thus a response made in trial *t* depends on the previous state in trial *t*−1. Rats showed the highest probability to transition to a premature response from another premature response. They were also more likely than chance to make a premature response after an NR trial and were less likely than chance to make a premature response after an R trial.

### Experiment 2

In this experiment, animals were tested using a longer ITI to increase the number of premature responses per session, thereby increasing the reliability of any effect of reward omission on premature responding. In addition, we investigated whether decreasing the time-out punishment period affected the likelihood of this response type. Reducing the time-out period may affect premature responding by increasing the reinforcement rate and by reducing the aversiveness of longer waiting times between trials associated with a premature response. Rats were tested on a continuous reinforcement schedule and on a partial reinforcement schedule (*p*(R) = 0.5) with a time-out of either 5 s or 1 s for both short (5 s) and long (7 s) ITIs.

**Figure 4.**
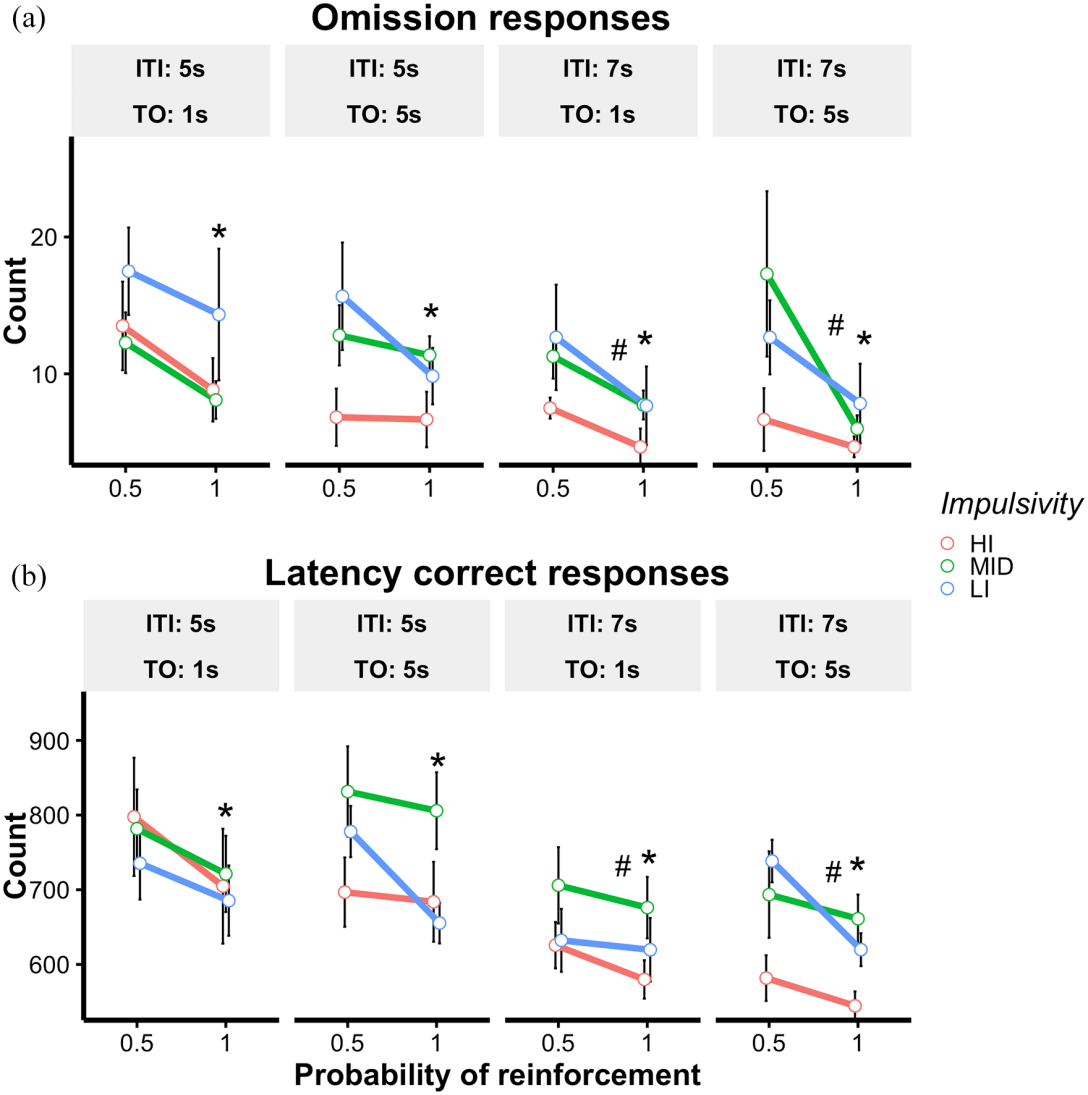
Experiment 2. Effects of ITI, *p*(R) and time-out on indices of motivation. (a) Omission responses, (b) latency to make a correct response (ms). *Statistically significant difference between or *p*(R) = 1 versus *p*(R) = 0.5, *p* < 0.05. ^#^Statistically significant difference between ITI 5 s and ITI 7 s, *p* < 0.05. LMEM was used for this analysis.

First, indices of motivation were analysed to explore how differences in reinforcement rate affected performance. Significant results are reported in Figure 4. A model including ITI (5 s and 7 s), time-out (5 s and 1 s), *p*(R) (*p*(R) = 0.5 and *p*(R) = 1) and impulsivity group (HI, MID and LI) showed that rats were significantly faster at making a correct response with *p*(R) = 1 than *p*(R) = 0.5 [F(1,138) = 12.23, *p* < 0.001] and when the ITI was 7 s compared to when it was 5 s [F(1,138) = 39.76, *p* < 0.001]. Similarly, the number of omissions was higher with *p*(R) = 0.5 compared to *p*(R) = 1 [F(1, 138) = 18.05, *p* < 0.001] and when the ITI was 5 s compared to 7 s [F(1,138) = 9.19 *p* = 0.003]. There was no significant difference in number of correct responses and latencies to restart trials across any of the manipulations.

**Figure 5.**
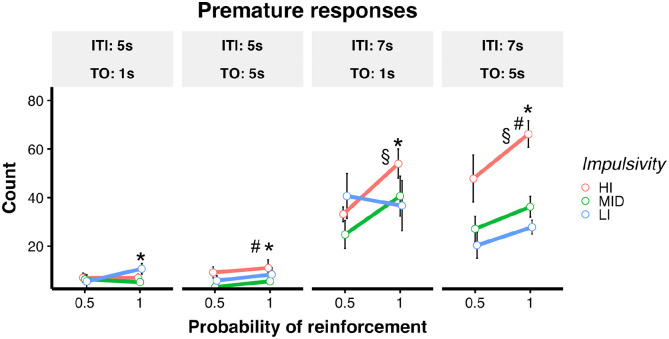
Experiment 2. Effects of ITI, *p*(R) and time-out on premature responses. *Statistically significant difference between or *p*(R) = 1 versus *p*(R) = 0.5, *p* < 0.05. ^#^Statistically significant difference between HI rats versus the other impulsivity groups when the time-out is 5 s, *p* < 0.05. ^§^Statistically significant difference between HI rats versus the other impulsivity groups when the ITI was 7 s, *p* < 0.05. LMEM was used for this analysis.

The effects of these manipulations on premature responses are shown in Figure 5. The model revealed a main effect of reward probability [F(1,138) = 12.73, *p* < 0.001], an interaction between ITI and impulsivity group [ITI × Group, F(2,138) = 3.98, *p* = 0.021], and an interaction between impulsivity group and time-out, [Time-Out × Group, F(2,138) = 3.94, *p* = 0.022]. Post hoc contrasts showed that rats made more premature responses during sessions with *p*(R) = 1 compared with *p*(R) = 0.5, across all manipulations (*p* < 0.001). In addition, HI rats made more premature responses than MID and LI rats during 7 s ITI sessions (*p* < 0.01 for all comparisons) and when the time-out was 5 s in duration compared with 1 s (*p* < 0.01 for all comparisons). Table S3 in the supplementary materials summarises significant correlations between number of premature responses and latency to make a correct response.

### Consequences of rewarded and non-rewarded trials on premature responses

**Figure 6.**
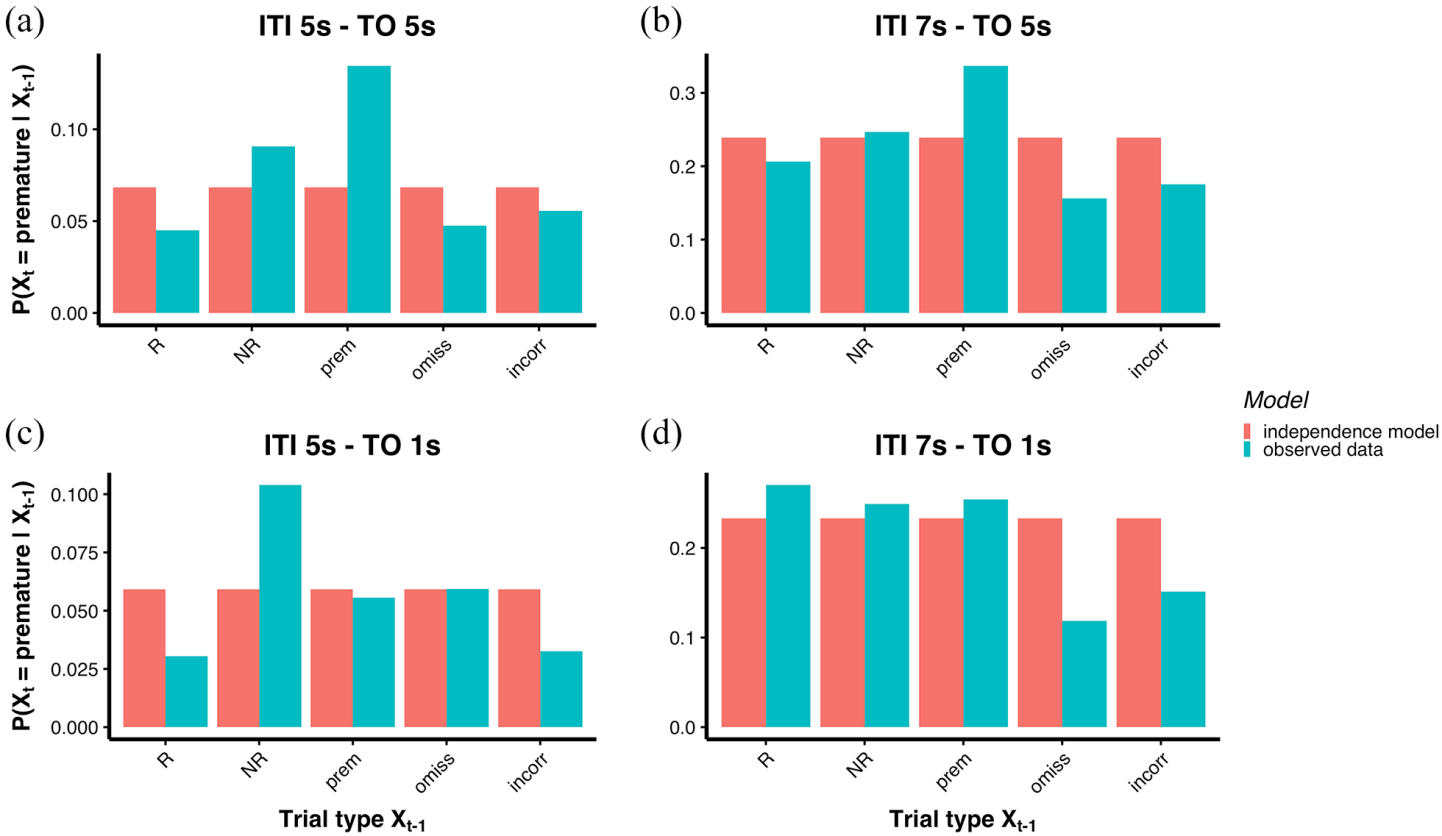
Experiment 2. Transition probabilities leading to a premature response (end state) in different conditions, *p*(R) = 0.5: (a) 5 s ITI and 5 s time-out. (b) 7 s ITI and 5 s time-out. (c) 5 s ITI and 1 s time-out. (d) 7 s ITI and 1 s time-out. *Y*-axis shows the probability to transition to a premature response as an end state (trial *t*). *X*-axis shows starting states (trial *t*−1). Red = independence model; Blue = observed data. TO = time-out.

**Figure 7.**
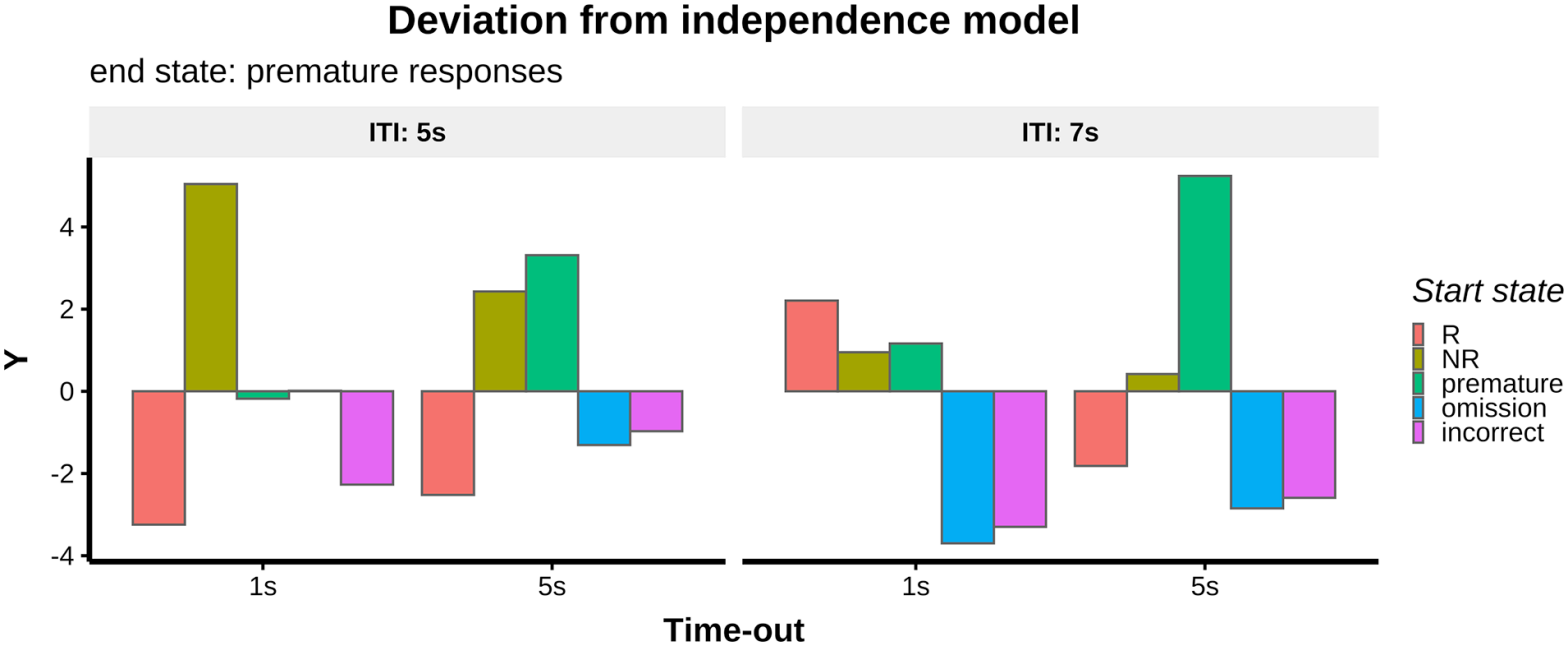
Experiment 2. Summary of the deviation from the independence model of transition probabilities leading to premature responses, across all experiments (*p*(R) = 0.5). *Y* = (O−E)/E^1/2^ was calculated for each start state ending in a premature response, O = observed data and E = expected frequencies under the independence model. The value 0 on the *x*-axis represents no deviation from the independence model.

The extent to which transition probabilities leading to a premature response deviated from the independence model, under different manipulations, is shown in Figure 6(a)–(d). Frequencies of one-step transitions and their deviation from the independence model are shown in the supplementary material (Figure S7). The *W* statistics and χ^2^ tests for each manipulation are summarised in Table 1. Replicating findings in Experiment 1, when the ITI was 5 s and the time-out punishment was 5 s, the largest deviation from the independence model was a higher-than-expected probability to transition to a premature response from a premature response (*Y* = 3.31) and a lower than expected probability to transition to a premature response from an R trial (*Y* = −2.52, see Figures 6(a) and **7**). Similar to Experiment 1, rats were also more likely to make a premature response after an NR trial (*Y* = 2.43, see Figures 6(a) and **7**), compared to what would be expected under the independence model. When the ITI was 7 s and the time-out punishment was 5 s, the largest deviation from the independence model was a higher-than-expected probability to transition to a premature response from a premature response (*Y* = 4.09, see Figures 6(b) and **7**). When the ITI was 5 s and the time-out was 1 s, the largest deviations from the independence model were a higher-than-expected probability to make a premature after an NR trial (*Y* = 5.04) and a lower-than-expected probability to make a premature after an R trial (*Y* = −3.24, see Figures 6(c) and **7**). When the ITI was 7 s and the time-out was 1 s, the largest deviations from the independence model were a lower-than-expected probability to make a premature after an omission response (*Y* = −3.70) and after an incorrect response (*Y* = −3.30, see Figures 6(d) and **7**). A summary of the deviation from the independence model (*Y*) for each manipulation is shown in Figure 7.

**Table 1.** Diagnostic tests for the first-order Markov chain model applied to sessions of the 5CSRTT with manipulations either to the ITI, the *p*(R) or the time-out.

	*p*(R) = 0.5	
	ITI = 5 s	ITI = 7 s
Time-out 1 s	*W* = 81.49, *p* < 0.001; χ^2^ = 41.15, *p* < 0.001	*W* = 118.50, *p* < 0.001; χ^2^ = 31.68, *p* < 0.025
Time-out 5 s	*W* = 130.00, *p* < 0.001; χ^2^ = 25.89, *p* < 0.001	*W* = 211, *p* < 0.001; χ^2^ = 45.76, *p* < 0.001

The *W* statistic is a diagnostic test to assess whether the matrix of transition probabilities considered is different from an independence model, which assumes no dependencies between states. The χ^2^ test applied to transition probabilities leading to a premature response narrows down the analysis performed by the *W* statistic and verifies whether transition probabilities leading to a premature response are different from a distribution in which there are no dependencies between states and rats are equally likely to make a premature response after any trial type. Tests show that for all analyses, performance on the five-choice serial reaction time task (5CSRTT) during both halves of the session, violated the independence model and were captured by a first-order Markov Chain model. Yellow shadowing indicates statistical significance.

Since HI rats made significantly more premature responses when the ITI was 7 s and when the time-out was 5 s, a first-order Markov chain model was separately applied to this subset of animals. These results did not reveal major differences in the transition probabilities that lead to a premature response between HI rats and the other two groups and are summarised in the supplementary material (Table S3, Figure S8).

In summary, rats were more motivated to perform the task during continuous reinforcement as indexed by a higher number of correct responses, faster response latencies and a decrease in omissions. Rats also made more premature responses during continuous reinforcement. HI rats made more premature responses than the other two groups when the ITI was increased from 5 s to 7 s and when the time-out was 5 s as opposed to 1 s. In relation to transition probabilities leading to a premature response, lengthening the ITI (7 s) did not alter the pattern observed in Experiment 1 (5 s ITI, 5 s time-out), specifically that a premature response is more likely to occur after a premature response. However, such a pattern was only observed when the time-out was 5 s. Shortening the time-out to 1 s led to an equal likelihood of a premature response following an R, NR and premature trials (when the ITI was 7 s) or to an increase in premature responses following an NR trial (when the ITI was 5 s).

## Discussion

These findings show that reinforcement rate as well as negative urgency play a role in modulating premature responses in the 5CSRTT. Specifically, at the macro-level, increasing the reinforcement rate increased the number of premature responses, supporting the sensitivity to reward hypothesis. However, at a micro-level of analysis, premature responses were more likely to occur after a correct but non-rewarded trial compared to a correct rewarded trial, supporting the frustration hypothesis. They were also likely to occur following another (non-reinforced/punished) premature response. This is also consistent with a possible role for negative urgency, although this form of premature response could have been due other factors. The likelihood of a premature response to follow either of these trial types depended on the duration of the time-out punishment.

Under a continuous reinforcement schedule (*p*(R) = 1), latencies to make a correct response decreased compared with partial reinforcement schedules (*p*(R) = 0.2; *p*(R) = 0.5; *p*(R) = 0.8). This finding is consistent with previous research and has been interpreted as indicating increased motivation to engage with the operant task (Hamid et al., 2016; Mohebi et al., 2019; Niv et al., 2007). Indeed, response vigour has been postulated to be controlled by the opportunity cost of not acting, with shorter latencies enabling individuals to maximise the amount of reward per unit of time (Niv et al., 2007). Concomitant with a shortening of latencies, there was also an increase in the number of premature responses (macro-level analysis) during continuous reinforcement. Importantly, in many manipulations, the latency to make a correct response correlated negatively with the number of premature responses. Since the latency to make a correct response could reflect task motivation (Robbins, 2002), these findings suggest that premature responses are modulated by incentive motivation processes.

The present results thus support the sensitivity to reward hypothesis in that premature responses increase concomitantly with the probability of reinforcement. On the contrary, these findings do not support the frustration hypothesis, which holds that frustration should ‘increase in strength as a function of non-rewarded trials’ (Amsel, 1992) and thus predicts an increase in premature responses during partial reinforcement. While macro*-*level analyses of performance did not reveal an effect of frustration on premature responses, it is still possible that the occurrence of premature responses in trial *t* is influenced by the frustration of a non-reward in trial *t*−1, be this an NR or a time-out punishment following the occurrence of incorrect, premature or omissions responses. For this reason, evaluation of performance on a trial-by-trial basis, that is, at the micro-level, was implemented on sessions with *p*(R) = 0.5, which have an equal distribution of R and NR trials and thus enable a direct assessment of the influence of frustration on premature responses. To test for dependencies between trial types, for example, whether premature responses were more likely to follow specific responses, a first-order Markov chain model was fit to all the possible transitions between trials and across animals. When the time-out was 5 s in duration and the ITI was either 5 s or 7 s, animals were more likely to transition to a premature response after an NR trial or another premature response and were much less likely than ‘chance’ to make a premature response after an R, incorrect or omitted trial. However, when the time-out was 1 s, rats were more likely to make a premature response either after an NR trial (when the ITI was 5 s) or after an R, NR or premature response (when the ITI was 7 s). A higher likelihood than chance to make a premature response after an NR trial supports the frustration hypothesis, which predicts an invigoration of behaviour following the omission of expected rewards. On the contrary, the lower likelihood of a premature response following R trials is consistent with post-consummatory inhibition (Seward et al., 1957) and challenges a simple version of the sensitivity to reward hypothesis, which would predict invigoration of behaviour following the receipt of reward.

Together with a higher-than-chance probability of making a premature response after an NR trial, rats exhibited a higher-than-chance probability of making a premature response after a previous premature response. The few instances in which this was not the case were when the time-out was reduced to 1 s. This points to a latent effect of the time-out interval, which scales with an increased propensity to make successive premature responses. This may be due to the fact that an increased time-out period increases the waiting interval, thus potentially augmenting urgency and the occurrence of a premature response in the upcoming trial. This interpretation is in line with evidence that trials ending in premature responses signal an earlier waiting period (Donnelly et al., 2015). In addition, an analysis of the temporal development of premature responses within-session shows that these tend to happen primarily in the first half of the session (see Figure S1 in the supplementary materials). This may be due to the heightened expectation of being rewarded during the start of the session, which may over-activate behaviour (Robbins and Everitt, 2007) and drive the occurrence of premature responses. It is possible that some of the premature responses occurring in series are driven by frustration; however, the negative urgency that would drive these responses would not result from the violation of an expected (and omitted) reward, since rats learn that premature responses are punished and thus should not expect to be rewarded for these responses.

Differences in premature responses across impulsivity groups were only evident when the requirement for waiting was challenged, that is, when the ITI was lengthened to 7 s, and when the time-out was kept at 5 s in duration. The former result is not surprising considering that HI and LI rats were selected based on premature responses made during long ITI trials (7 s and 9 s) of two sessions of a vITI paradigm. This was done, in accordance with previous research (Caprioli et al., 2013; Dalley et al., 2007), because long ITIs are known to challenge waiting impulsivity and thus reveal a vulnerability for an inability to withhold a response (Bari et al., 2008). It is noteworthy that the impulsivity subgroups also differed between each other when the time-out was kept at 5 s. This was due to the fact that LI and MID rats made fewer premature responses when the time-out was 5 s compared to 1 s. This effect could be mediated by increased reinforcement rates due to shorter time-outs, which increase behavioural activation and consequently premature responses. LI and MID rats are perhaps more sensitive to such indirect changes in reinforcement rate. Nonetheless, the lack of an interaction between impulsivity phenotype and reinforcement rate on all indices of performance on the 5CSRTT suggests that all impulsivity groups, including HI rats, were equally sensitive to decrements in motivation and the effect that this had on premature responses. Equally, analyses at the micro-level did not suggest HI rats to be more or less susceptible to negative urgency. Markov-chain models fitted specifically on HI rats separately from the other two groups cannot provide conclusive evidence on whether trial history prior to a premature response is different between impulsivity groups because there were too few HI rats for reliable statistical power. However, preliminary evidence reported in the supplementary material (Figure S8) does not point to substantial differences in the transition probabilities that lead to a premature response existing between HI rats and the other two impulsivity groups. Thus, with regards to premature responses, HI rats are subject to the same modulatory effects of reinforcement probability and negative urgency as the other two impulsivity groups. However, HI rats differ significantly from the other groups when the requirement for waiting is challenged, resulting in an increased propensity for premature responses.

Taken together, these experiments suggest that premature responses are broadly influenced by manipulations that affect motivation to perform a task, in favour of the sensitivity to reward hypothesis, and of reward omission in favour of the frustration hypothesis. These findings are in line with observations in experimental animals (Hamid et al., 2016; Judice-Daher et al., 2012; Mohebi et al., 2019) and humans (Cools et al., 2005; Dixon et al., 2013; Whiteside and Lynam, 2001) showing that both positive reinforcement and the unexpected omission of positive reinforcement can activate behaviour, driving rapid responding. Importantly, in humans, impulsivity associated both with negative urgency (Jia et al., 2021; Latzman et al., 2013; Magid and Colder, 2007) and with sensitivity to reward (Bjork et al., 2008) have been linked to maladaptive behaviour such as problematic alcohol and substance use. This study shows that these factors may also underlie impulsive responding in experimental approaches to study addiction in animals (Belin et al., 2008; Dalley et al., 2007). Collectively, these findings highlight the importance of understanding the multi-factorial nature of impulsivity and their underlying neural and psychological substrates to inform more specific interventions in clinical disorders of impulsivity.

## Supplemental Material

sj-docx-1-bna-10.1177_23982128221102256 – Supplemental material for Dissociating reward sensitivity and negative urgency effects on impulsivity in the five-choice serial reaction time task Click here for additional data file. Supplemental material, sj-docx-1-bna-10.1177_23982128221102256 for Dissociating reward sensitivity and negative urgency effects on impulsivity in the five-choice serial reaction time task by Chiara Toschi, Mona El-Sayed Hervig, Thiago Burghi, Torben Sell, Matthew Dominic Lycas, Parisa Moazen, Li Huang, Ulrik Gether, Trevor W. Robbins and Jeffrey W. Dalley in Brain and Neuroscience Advances
