# Supplementary material for: Dissociating reward sensitivity and negative urgency effects on impulsivity in the five-choice serial reaction time task
Source: Brain Neurosci Adv. 2022 Jun 14;6:23982128221102256. doi: 10.1177/23982128221102256 (PMC9201310; doi:10.1177/23982128221102256)
Supplement: sj-docx-1-bna-10.1177_23982128221102256 – Supplemental material for Dissociating reward sensitivity and negative urgency effects on impulsivity in the five-choice serial reaction time task [file sj-docx-1-bna-10.1177_23982128221102256.docx]

**Supplementary Materials**

**Methods**

#### Temporal development of premature responses


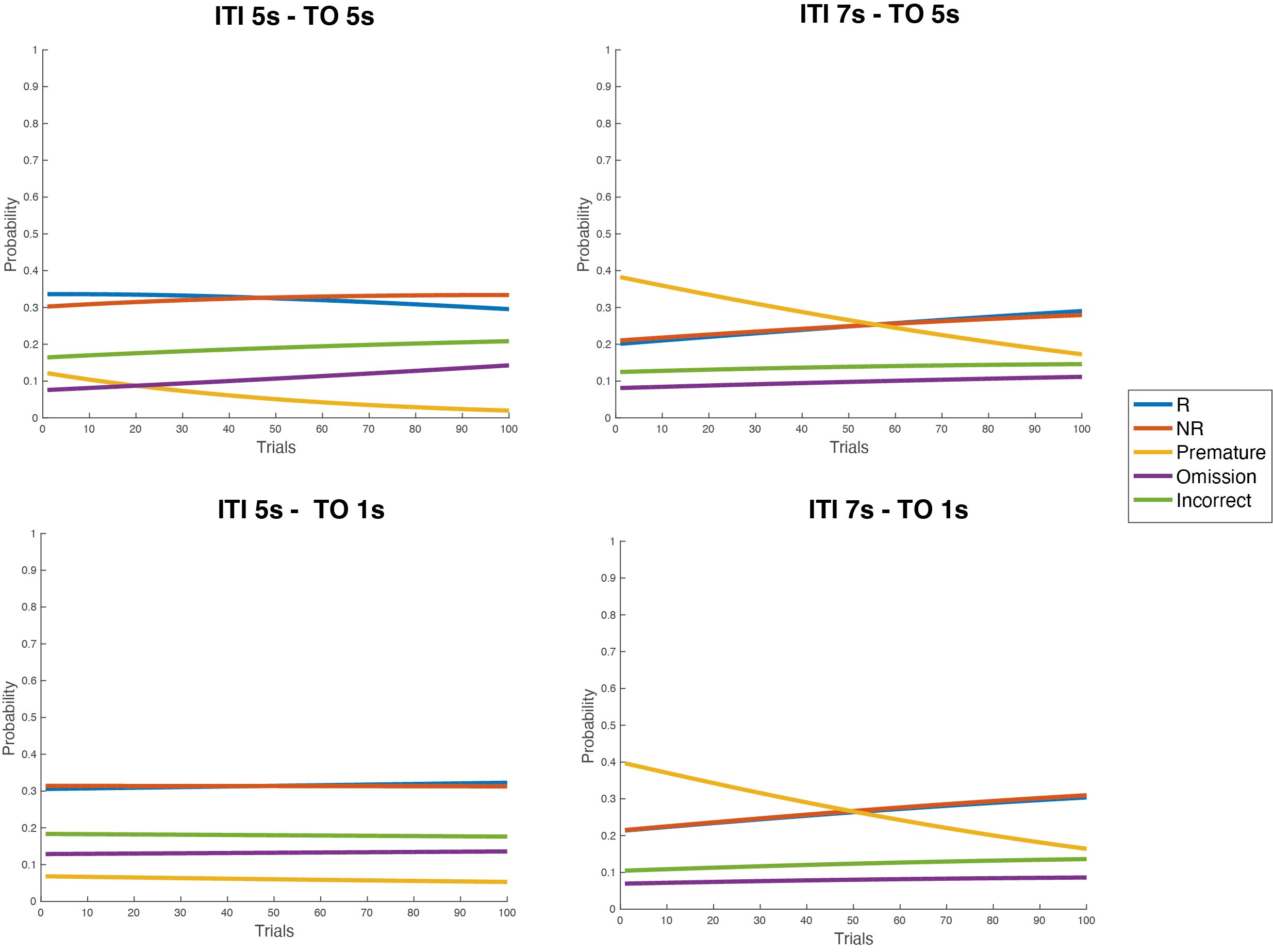


Figure S1. Experiment 2: Cohort 1. Overview of the temporal development, within-session, of the likelihood of making either correct rewarded (R), correct non-rewarded (NR), premature, omissions or incorrect responses during a session with p(R)=0.5. Multinomial logistic regressions were used to represent this data.


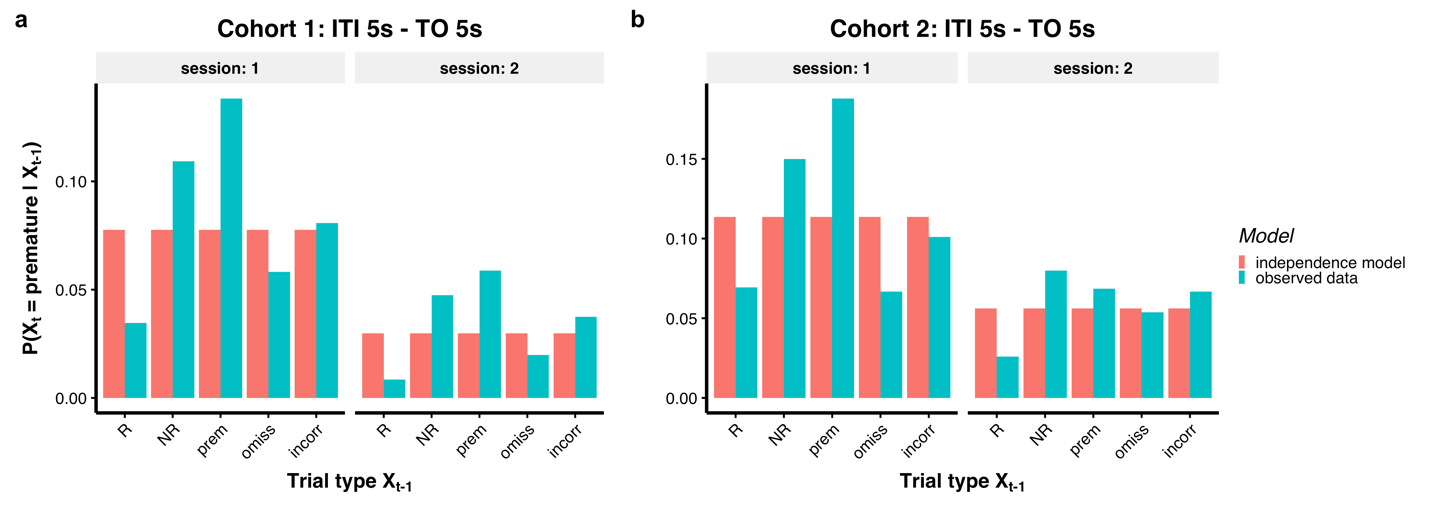


**Figure S2.** **Experiment 1: Cohort 1 and 2.** Transition probabilities leading to a premature response (end state) for the first half and second half of each session of Experiment 1 for both Cohort 1 and 2, p(R)=0.5. **(a)** Cohort 1 (N=23) 5 s ITI and 5 s time-out. **(b)** Cohort 2 (N=36) 5 s ITI and 5 s time-out. Y-axis shows the probability to transition to a premature response as an end state (trial t). X-axis shows starting states (trial t-1). Red = independence model; Blue = observed data. TO = time-out.


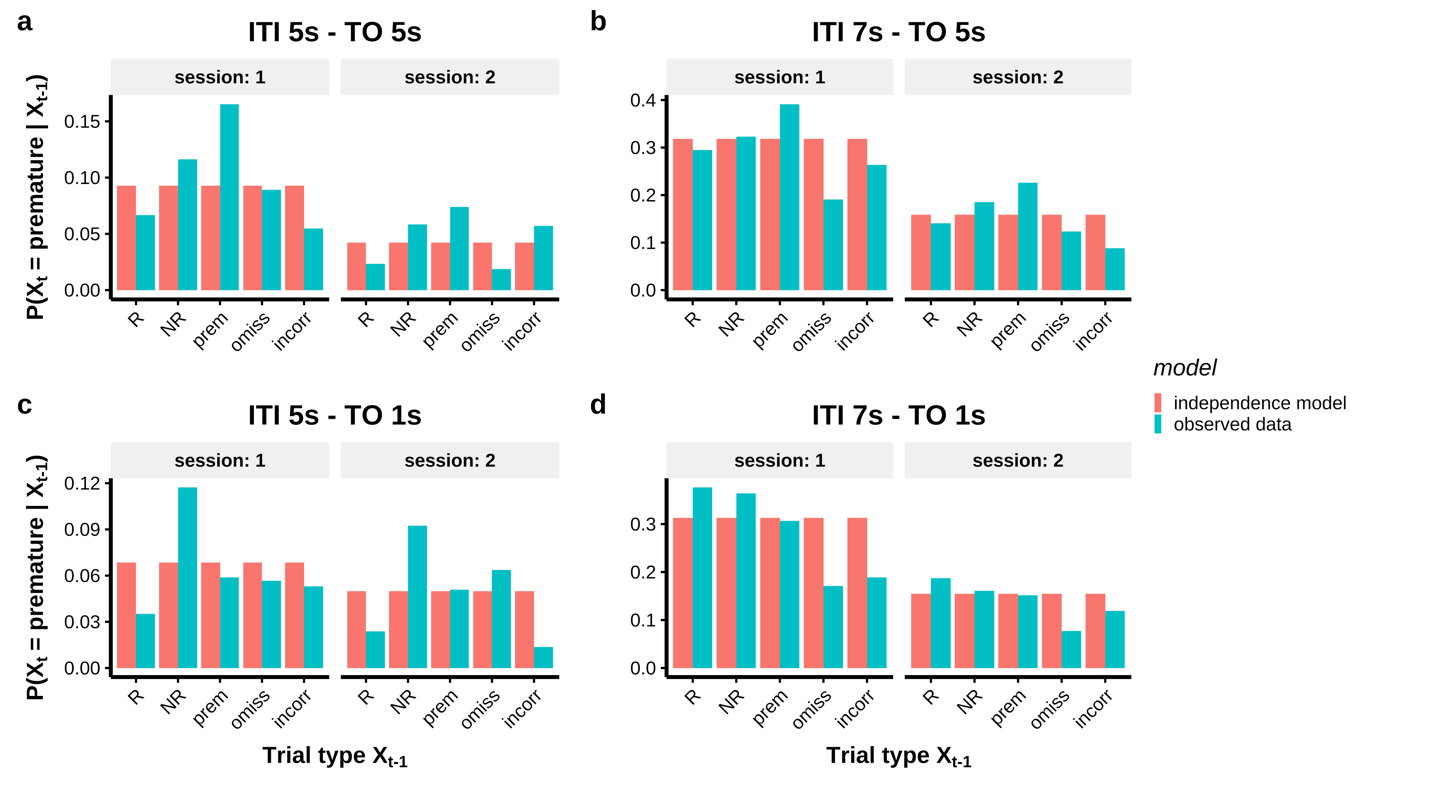


**Figure S3.** **Experiment 2: Cohort 1.** Transition probabilities leading to a premature response (end state) for the first half and second half of each session of each manipulation, p(R)=0.5. **(a)** 5 s ITI and 5 s time-out. **(b)** 7 s ITI and 5 s time-out. **(c)** 5 s ITI and 1 s time-out. **(d)** 7 s ITI and 1 s time-out. Y-axis shows the probability to transition to a premature response as an end state (trial t). X-axis shows starting states (trial t-1). Red = independence model; Blue = observed data. TO = time-out.

|  | **Session: first half** | **Session: second half** |
| --- | --- | --- |
| **ITI 5 s - time out 5 s,**  **(Experiment 1)** | Cohort 1:  W=49.32, p<0.01  X^2^= 18.80, p<0.01  Cohort 2:  W= 96.54, p<0.01  X^2^= 33.32, p<0.01 | Cohort 1:  W= 41.06, p<0.01  X^2^= 11.10, p<0.05  Cohort 2:  W= 49.58, p<0.01  X^2^= 16.59, p<0.05 |
| **ITI 5 s - time out 5 s,**  **(Experiment 2)** | Cohort 1:  W=55.35, p<0.01  X^2^= 14.74, p<0.02 | Cohort 1:  W=76.16, p<0.01  X^2^= 10.17, p>0.05 |
| **ITI 7 s - time out 5 s**  **(Experiment 2)** | W= 90.65, p<0.01  X^2^= 16.43, p<0.01 | W= 116.90, p<0.01  X^2^= 16.39, p<0.01 |
| **ITI 5 s - time out 1 s**  **(Experiment 2)** | W= 41.98, p<0.01  X^2^=20.14, p<0.01 | W= 53.14, p<0.01  X^2^= 24.81, p<0.01 |
| **ITI 7 s - time out 1 s**  **(Experiment 2)** | W=79.69, p<0.01  X^2^=23.70, p<0.01 | W=56.53, p<0.01  X^2^=9.96, p>0.05 |

**Table S1. W and X^2^ statistic for the diagnostic tests applied to a first-order Markov chain for the first and second half of the 5CSRTT sessions, for each manipulation** (p(R)=0.5 in all manipulations). The W statistic is a diagnostic test to assess whether the matrix of transition probabilities considered is different from an independence model, which assumes no dependencies between states. The X^2^ test applied to transition probabilities leading to a premature response narrows down the analysis performed by the W statistic and verifies whether transition probabilities leading to a premature response are different from a distribution in which there are no dependencies between states and rats are equally likely to make a premature response after any trial type. Tests show that for all analyses, performance on the 5CSRTT during both halves of the session, violated the independence model and were captured by a first-order Markov Chain model. The few instances in which this was not the case were for the second half of the session (1) when ITI was 5 s and time out 5 s and (2) when the ITI was 7 s and the timeout was 1 s. Yellow shadowing indicates statistical significance both of the W statistic and of the X^2^ statistic.

# Results

# Experiment 1 - Effects of partial reinforcement on premature responses

# Cohort 2

Similarly to Cohort 1, p(R) had an effect on number of correct and omission responses for Cohort 2. As shown in Table S2, reinforcement rate had an effect on number of correct responses [F(3,99)=5.58, p=0.001]. Animals made less correct responses in p(R)=0.2 compared to p(R)=1 (p=0.004), p(R)=0.8 (p=0.003) and p(R)=0.5 (trend level of significance, p=0.057). Similarly to Cohort 1, p(R) also affected number of omissions during a session, [F(2.99)=20.29, p<0.001]. Specifically, animals made more omission responses in p(R)=0.2 compared to p(R)=1, p(R)=0.8 and p(R)=0.5 (p<0.001 for all comparisons). Animals also made more omissions during p(R)=0.5 compared to p(R)=1 (p=0.024). Latency to make a correct response was affected by p(R), [F(3,99)=11.06, p<0.001]. Animals were faster at making a correct response in p(R)=1 compared to p(R)=0.5 (p=0.023) and p(R)=0.2 (p<0.001). Animals were also faster at making a correct response in p(R)=0.8 compared to p(R)=0.2 (p<0.001). Similar to Cohort 1, latency to make a correct response was analysed as a function of trial outcome (R vs NR responses) to check whether animals could predict whether the upcoming trial would be rewarded or not when making a correct response. There was no effect of outcome on latency to make a correct response [F(1,165)=0.85, p=0.358].

| **Cohort 2** | **p(R)=0.2** | **p(R)=0.5** | **p(R)=0.8** | **p(R)=1** |
| --- | --- | --- | --- | --- |
| **Correct responses** | 68.27 (2.33)*^$^ | 74.03 (1.25) | 76.72 (1.44) | 76.75 (1.33) |
| **Omission responses** | 16.17 (1.82)*^$^^ | 9.36 (1.16)* | 6.36 (0.98) | 4.81 (0.71) |
| **Latency correct responses (ms)** | 769.97 (25.90)*^$^ | 723.89 (23.36)* | 667.01 (19.28) | 649.94 (24.34) |
| **Latency to restart a trial following time-out (ms)** | 6165.45 (636.97)*^$^^ | 4497.46 (441.64) | 3874.65 (344.96) | 3833.48 (311.274) |

Table S2 Cohort 2. Effects of p(R) on number of correct and omission responses, latency to make a correct response and to re-start a trial following a time-out. Mean and (SE). *statistical significant difference with p(R)=1 p<0.05  ^$^statistical significant difference with p(R)=0.8 p<0.05. ^statistical significant difference with p(R)=0.5 p<0.05. LMEM were used for these analyses.


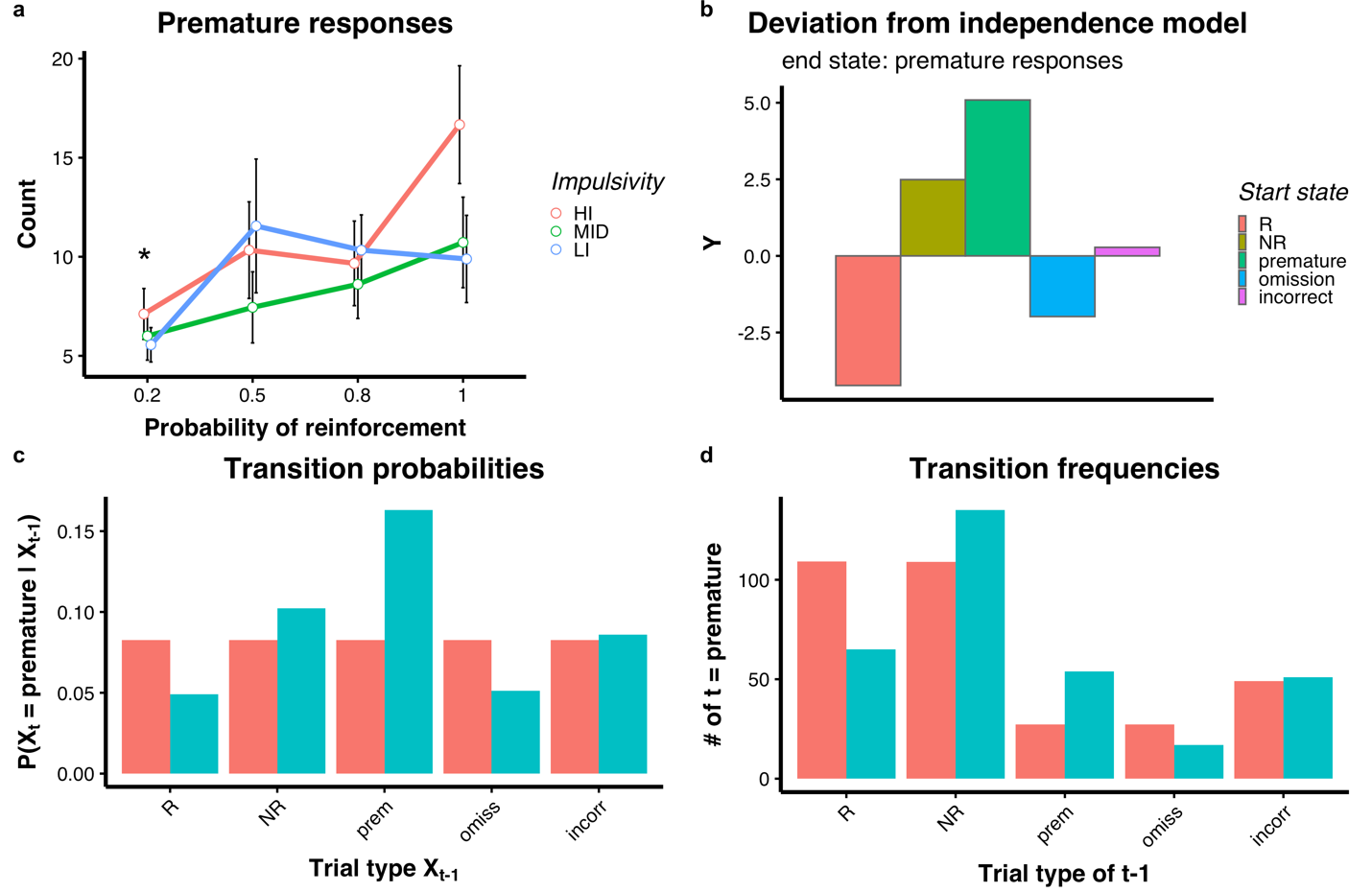


**Figure S4. Experiment 1 - Cohort 2.** **(a)** Effects of reinforcement rate on premature responses (Mean and standard error, SE); *statistical difference between p(R)=0.2 and p(R)=1, p<0.05. LMEM was used for this analysis; **(b)** Deviation from the independence model of transition probabilities leading to premature responses for Experiment 1 of Cohort 2 during p(R)=0.5. Y=(O-E)/E^1/2^ was calculated for each start state ending in a premature response, O = observed data and E = expected data under the assumption of the independence model. The value 0 on the x-axis represents no deviation from the independence model. **(c)** Transition probabilities leading to a premature response (end state) for p(R)=0.5. Y-axis shows the probability to transition to a premature response as an end state (trial t). X-axis shows starting states (trial t-1). **(d)** Frequencies of one-step transitions leading to a premature response (end state) for p(R)=0.5. Y-axis shows how often a premature response was an end state (trial t). X-axis shows starting states (trial t-1). Red = independence model; Blue = observed data.

**Figure S4a** shows the effects of p(R) on premature responses. There was a significant effect of p(R) on premature responses for Cohort 2, [F(3,99)=3.83, p=0.012], with animals making fewer premature responses when reinforcement was delivered with a probability of p(R)=0.2 compared to p(R)=1 (p=0.012). In all reinforcement rates, except p(R)=0.2, there was a negative correlation between the number of premature responses and the latency to make a correct response, thus for p(R)=0.5: *r=-0.54, p<0.001*; p(R)=0.8: *r=-0.39, p=0.018*; p(R)=1: *r=-52, p=0.001*.

# Consequences of a rewarded or non-rewarded trial on premature responses

# Cohort 2

Similar to Cohort 1, the Markov chain model was fit to the performance data of Cohort 2 and was tested for a violation of the independence model. The independence model was violated as shown by a W statistic of 130.06 with significance level p<0.001. A chi-square test on the frequencies of one-step transitions leading to a premature response showed that these are significantly different from the distribution that would be expected if there were no dependencies between trials, X^2^=54.01 p<0.001. **Figure S4c** and **d** show how the transition probabilities and frequencies of one-step transitions, respectively, leading to a premature response deviated from the independence model. The biggest deviations from the independence model were a lower-than-expected probability to transition to a premature response from a R trial (Y=-4.23) and a higher-than-expected probability to transition to a premature response from a premature response (Y=5.09, see **Figure S4b**). Similar to Cohort 1,. rats in Cohort 2 were also more likely to make a premature response after a NR trial (Y = 2.49, see **Figure S4b**), compared to what would be expected under the independence model.

# Consequences of a rewarded or non-rewarded trial on premature responses for all reinforcement schedules


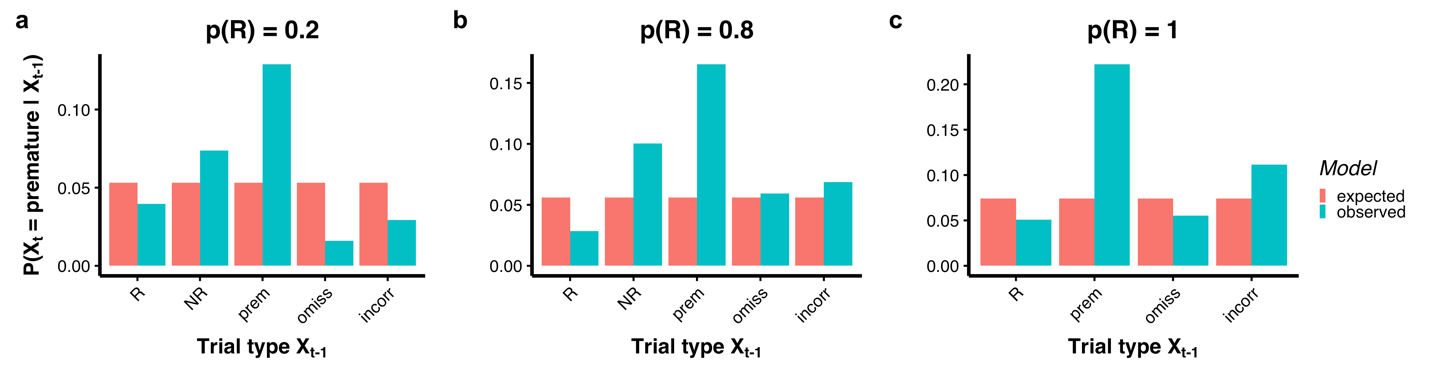


Figure S5. Experiment 1: Cohort 1. Transition probabilities leading to a premature response (end state) for reinforcement rates (a) p(R)=0.2; (b) p(R)=0.8; (c) p(R)=1. Y-axis shows the probability to transition to a premature response as an end state (trial t). X-axis shows starting states (trial t-1). Red = independence model; Blue = observed data. For all reinforcement schedules the W statistic and X^2^ statistic revealed a significant deviation from the independence model (p<0.01).


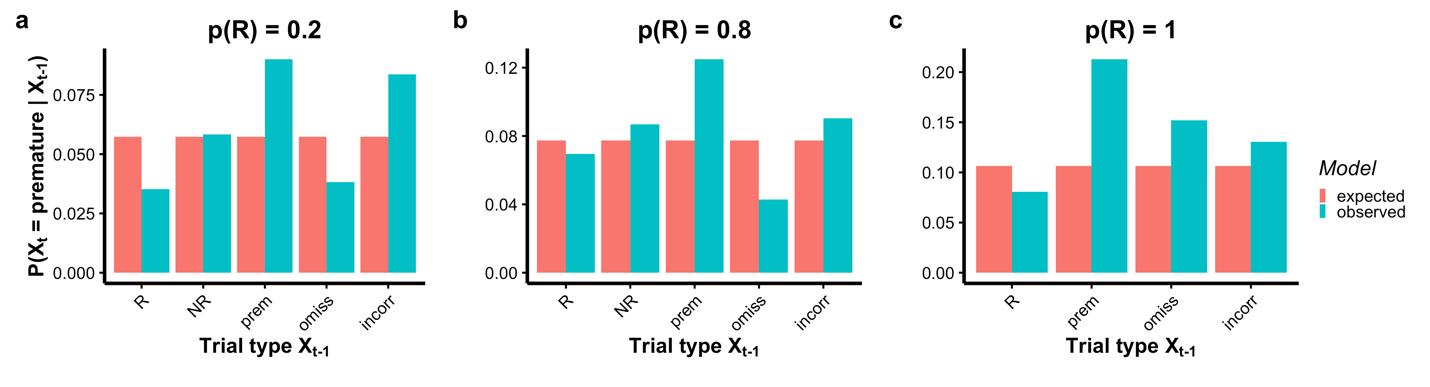
Figure S6. Experiment 1: Cohort 2. Transition probabilities leading to a premature response (end state) for reinforcement rates (a) p(R)=0.2; (b) p(R)=0.8; (c) p(R)=1. Y-axis shows the probability to transition to a premature response as an end state (trial t). X-axis shows starting states (trial t-1). Red = independence model; Blue = observed data. For all reinforcement schedules the W statistic and X^2^ statistic revealed a significant deviation from the independence model (p<0.01).

# Experiment 2

|  | **Pr=1** | | **Pr=0.5** | |
| --- | --- | --- | --- | --- |
|  | **ITI 5 s** | **ITI 7 s** | **ITI 5 s** | **ITI 7 s** |
| **Time-out 1 s** | *r=-0.44,p=0.037* |  |  | *r=-0.69,p<0.001* |
| **Time-out 5 s** |  | *r=-0.51 p=0.016* |  |  |

**Table S3. Experiment 2: Cohort 1.** Correlations between premature responses and latency to make a correct response for each manipulation of Experiment 2. Only significant correlations are reported.


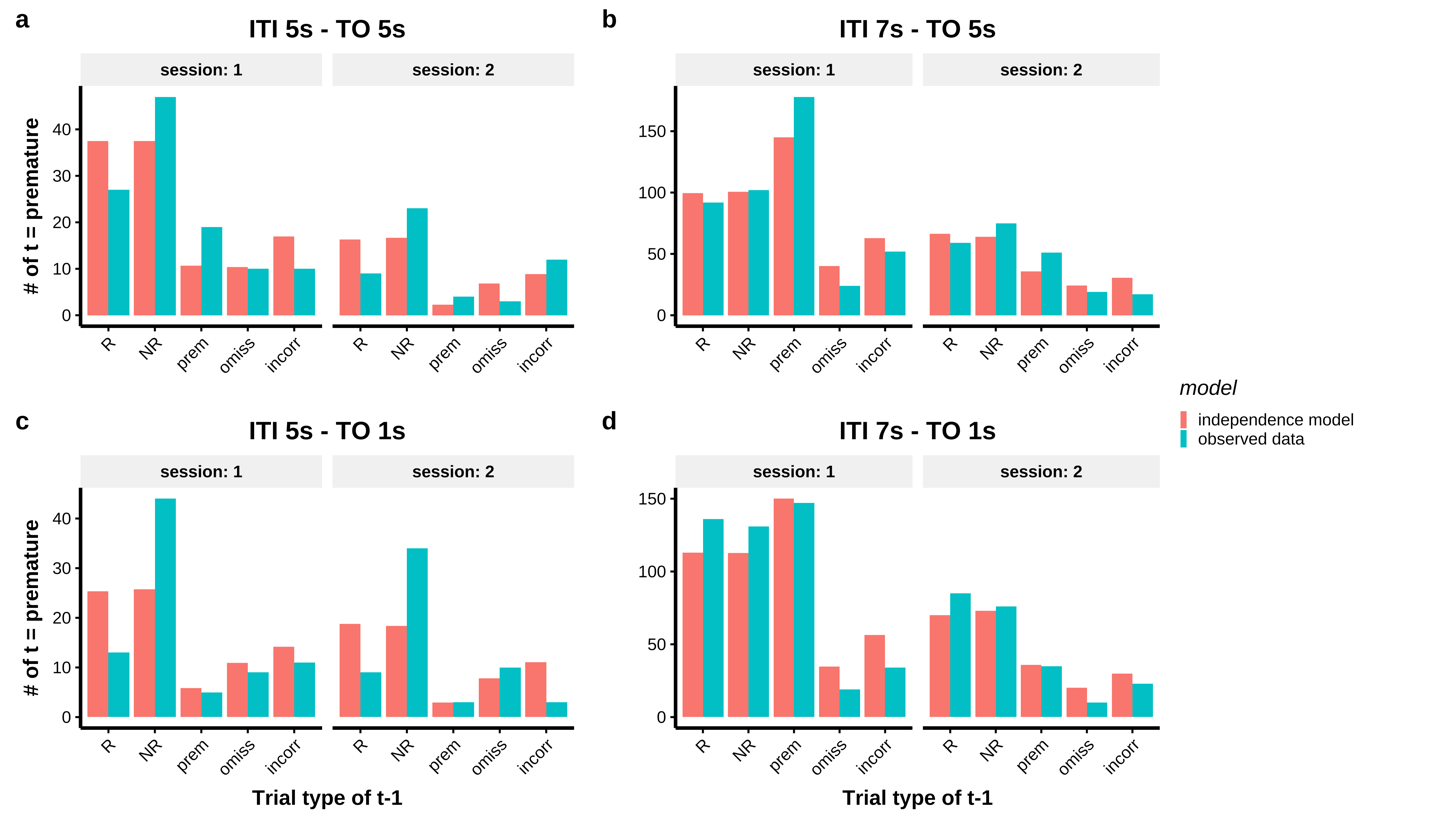


Figure S7. Experiment 2: Cohort 1. Frequencies of one-step transitions leading to a premature response (end state) for each manipulation of Experiment 2, p(R)=0.5: (a) 5 s ITI and 5 s time-out; (b) 7 s ITI and 5 s time-out; (c) 5 s ITI and 1 s time-out; (d) 7 s ITI and 1 s time-out. Y-axis shows how often a premature response was an end state (trial t). X-axis shows starting states (trial t-1). Red = independence model; Blue = observed data. TO = time-out.

# Consequences of a rewarded or non-rewarded trial on premature responses on two impulsivity groups

The transition probabilities between trial types were modelled with a Markov chain, to test whether animals were more likely to make a premature response after a frustrative event, such as a NR trial. Because HI rats made significantly more premature responses than the other groups when the ITI was 7 s and when the time-out was 5 s, two separate Markov chain models were fitted, one for HI rats and one for LI and MID rats combined for all manipulations. This was aimed at exploring whether impulsive responding in HI rats is predicted by a different trial history. **Table S4** shows the diagnostic W statistic for the Markov chain model applied to performance in each manipulation and the X^2^ test for the transition probabilities leading to a premature response in each manipulation. Most analyses applied to HI rats did not reach significance for both the W and the X^2^ test likely due to the lower number of HI rats compared to the other two groups. Thus data from a greater number of HI rats is needed to draw any conclusions on whether the pattern of transition probabilities leading to a premature response is different for HI rats compared to MID and LI rats. Preliminary evidence of such transitions plotted in **Figure S8**, however, does not suggest consistent differences between HI rats and the other two groups in the trial history preceding a premature response.


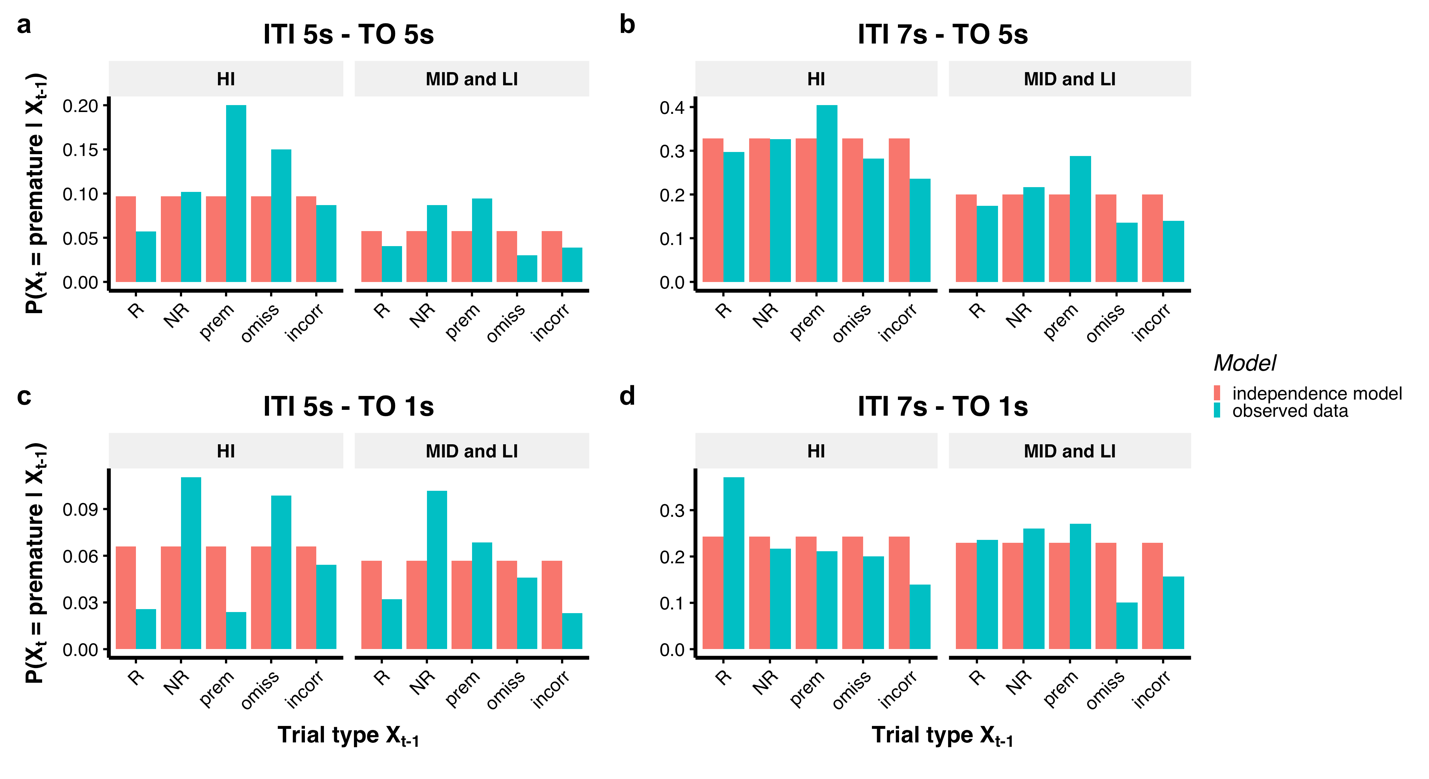


**Figure S8 Experiment 2: Cohort 1.** Transition probabilities leading to a premature response (end state) for HI rats and for MID and LI rats combined, for each manipulation of Experiment 2, p(R)=0.5: **(a)** 5 s ITI and 5 s time-out; **(b)** 7 s ITI and 5 s time-out; **(c)** 5 s ITI and 1 s time-out; **(d)** 7 s ITI and 1 s time-out. Transition probabilities leading to a premature response (end state). Y-axis shows the probability to transition to a premature response as an end state (trial t). X-axis shows starting states (trial t-1). Red = independence model; Blue = observed data. TO = time-out.

|  | **HI rats** | | **MID and LI rats** | |
| --- | --- | --- | --- | --- |
|  | **ITI 5 s** | **ITI 7 s** | **ITI 5 s** | **ITI 7 s** |
| Time-out 1 s | W=23.62, p>0.05  X^2^=13.18, p<0.05 | W=51.89, p<0.001  X^2^=21.42, p<0.001 | W = 80.31, p<0.001  X^2^= 32.73, p<0.05 | W=94.42, p<0.001  X^2^=26.82, p<0.001 |
| Time-out 5 s | W = 54.83, p<0.05  X^2^=11.89, p<0.05 | W=56.42, p<0.001  X^2^=9.58, p>0.05 | W=91.98, p<0.001  X^2^=18.93, p<0.001 | W=152.51, p<0.001  X^2^=27.72, p<0.001 |

Table S3. Diagnostic tests for the first-order Markov chain models applied separately for HI rats and MID and LI rats (combined) to sessions of the 5CSRTT with manipulations either to the ITI, the p(R) or the time-out, p(R)=0.5. The W statistic is a diagnostic test to assess whether the matrix of transition probabilities considered is different from an independence model, which assumes no dependencies between states. The X^2^ test applied to transition probabilities leading to a premature response narrows down the analysis performed by the W statistic and verifies whether transition probabilities leading to a premature response are different from a distribution in which there are no dependencies between states and rats are equally likely to make a premature response after any trial type. Tests show that for some analyses, performance of HI rats on the 5CSRTT either did not violate the independence model (ITI 5 s + time-out 1 s), and is thus not captured by a first-order Markov chain, or, where this was the case, the transition probabilities leading to a premature response were not significantly different from what would be expected in a condition where there are no dependencies between trials (ITI 7 s + time-out 5 s). On the contrary, performance of MID and LI rats combined was always captured by a first-order Markov chain. The difference in the significance of the tests between these two groups is likely due to the fact that HI rats are too few animals to detect a significant effect. Yellow shadowing indicates statistical significance.

| Experiment | Fixed factor | Level of Fixed factor | Coefficient Estimates | SE | t |
| --- | --- | --- | --- | --- | --- |
| Experiment 1 – Cohort 1 | Correct responses | p(R)=0.5 | 0.52 | 0.31 | 1.68 |
|  |  | p(R)=0.8 | 0.68 | 0.31 | 2.23 |
|  |  | p(R)=1 | 0.62 | 0.31 | 2.04 |
|  | Omission responses | p(R)=0.5 | -1.28 | 0.51 | -2.52 |
|  |  | p(R)=0.8 | -2.01 | 0.51 | -3.95 |
|  |  | p(R)=1 | -1.19 | 0.51 | -2.35 |
|  | Latency to make a correct response | p(R)=0.5 | -0.05 | 0.08 | -0.71 |
|  |  | p(R)=0.8 | -0.07 | 0.08 | -0.92 |
|  |  | p(R)=1 | -0.14 | 0.08 | -1.92 |
|  | Latency to start a trial (after a time-out) | p(R)=0.5 | -0.36 | 0.24 | -1.52 |
|  |  | p(R)=0.8 | -0.66 | 0.24 | -2.75 |
|  |  | p(R)=1 | -0.37 | 0.24 | -1.48 |

Table S4. Estimates of the fixed effects of the LMEM ran for Experiment 1 – Cohort 1. The estimates reported are: the slope or coefficient of the fixed effect predictors; the standard error (SE) associated with the slope, and the t value, which is simply the coefficient estimate divided by the SE. Estimates are reported only for those fixed factors that are found to statistically significant through the anova method.

| Experiment | Fixed factor | Level of Fixed factor | Coefficient Estimates | SE | t |
| --- | --- | --- | --- | --- | --- |
| Experiment 1 – Cohort 2 | Correct responses | p(R)=0.5 | 0.20 | 0.24 | 0.84 |
|  |  | p(R)=0.8 | 0.10 | 0.24 | 0.41 |
|  |  | p(R)=1 | 0.27 | 0.24 | 1.19 |
|  | Omission responses | p(R)=0.5 | -1.61 | 0.47 | -3.43 |
|  |  | p(R)=0.8 | -1.63 | 0.47 | -3.48 |
|  |  | p(R)=1 | -2.21 | 0.47 | -4.72 |
|  | Latency to make a correct response | p(R)=0.5 | -0.01 | 0.01 | -1.04 |
|  |  | p(R)=0.8 | -0.02 | 0.01 | -2.02 |
|  |  | p(R)=1 | -0.01 | 0.01 | -2.28 |
|  | Latency to start a trial (after a time-out) | p(R)=0.5 | -0.62 | 0.19 | -3.19 |
|  |  | p(R)=0.8 | -0.65 | 0.19 | -3.38 |
|  |  | p(R)=1 | -0.75 | 0.19 | -3.88 |
|  | Premature responses | p(R)=0.5 | 0.56 | 0.52 | 1.08 |
|  |  | p(R)=0.8 | 0.44 | 0.52 | 0.86 |
|  |  | p(R)=1 | 1.41 | 0.52 | 2.71 |

Table S5. Estimates of the fixed effects of the LMEM ran for Experiment 1 – Cohort 2. The estimates reported are: the slope or coefficient of the fixed effect predictors; the standard error (SE) associated with the slope, and the t value, which is simply the coefficient estimate divided by the SE. Estimates are reported only for those fixed factors that are found to statistically significant through the anova method.

| **Experiment** | **Fixed factor** | **Level of Fixed factor** | **Coefficient Estimates** | **SE** | **t** |
| --- | --- | --- | --- | --- | --- |
| **Experiment 2 – Cohort 1** | **Latency to make a correct response** | ITI 7 s | -0.22 | 0.08 | -2.80 |
|  |  | p(R)=1 | -0.14 | 0.08 | -1.67 |
|  | **Omission responses** | ITI 7 s | -0.83 | 0.54 | -1.54 |
|  |  | p(R)=1 | -0.72 | 0.54 | -1.34 |
|  | **Premature responses** | p(R)=1 | 0.01 | 0.71 | 0.02 |
|  |  | impulsivityMID:ITI7s | -0.90 | 0.88 | -1.02 |
|  |  | impulsivityLI:ITI7s | 0.80 | 1.00 | 0.80 |
|  |  | impulsivityMID:TO5s | -0.80 | 0.88 | -0.90 |
|  |  | impulsivityLI:TO5s | -0.54 | 1.00 | -0.59 |

Table S6. Estimates of the fixed effects of the LMEM ran for Experiment 2 – Cohort 1. The estimates reported are: the slope or coefficient of the fixed effect predictors; the standard error (SE) associated with the slope, and the t value, which is simply the coefficient estimate divided by the SE. Estimates are reported only for those fixed factors that are found to statistically significant through the anova method.

|  | Pr=1 | | Pr=0.5 | |
| --- | --- | --- | --- | --- |
|  | ITI 5 s | ITI 7 s | ITI 5 s | ITI 7 s |
| Time-out 1 s | *r=-0.44,p=0.037* |  |  | *r=-0.69,p<0.001* |
| Time-out 5 s |  | *r=-0.51 p=0.016* |  |  |
